# Mechanisms of Dominant Electrophysiological Features of Four Subtypes of Layer 1 Interneurons

**DOI:** 10.1523/JNEUROSCI.1876-22.2023

**Published:** 2023-05-03

**Authors:** John Hongyu Meng, Benjamin Schuman, Bernardo Rudy, Xiao-Jing Wang

**Affiliations:** ^1^Center for Neural Science, New York University, New York, New York 10003; ^2^Neuroscience Institute, Department of Neuroscience and Physiology, New York University, New York, New York 10016; ^3^Department of Anesthesiology, Perioperative Care, and Pain Medicine, New York University, New York, New York 10016

**Keywords:** electrophysiology, interneurons, irregularity, layer 1, single-cell modeling, VIP cells

## Abstract

Neocortical layer 1 (L1) consists of the distal dendrites of pyramidal cells and GABAergic interneurons (INs) and receives extensive long-range “top-down” projections, but L1 INs remain poorly understood. In this work, we systematically examined the distinct dominant electrophysiological features for four unique IN subtypes in L1 that were previously identified from mice of either gender: Canopy cells show an irregular firing pattern near rheobase; neurogliaform cells are late-spiking, and their firing rate accelerates during current injections; cells with strong expression of the α7 nicotinic receptor (α7 cells), display onset (rebound) bursting; vasoactive intestinal peptide (VIP) expressing cells exhibit high input resistance, strong adaptation, and irregular firing. Computational modeling revealed that these diverse neurophysiological features could be explained by an extended exponential-integrate-and-fire neuron model with varying contributions of a slowly inactivating K^+^ channel, a T-type Ca^2+^ channel, and a spike-triggered Ca^2+^-dependent K^+^ channel. In particular, we show that irregular firing results from square-wave bursting through a fast-slow analysis. Furthermore, we demonstrate that irregular firing is frequently observed in VIP cells because of the interaction between strong adaptation and a slowly inactivating K^+^ channel. At last, we reveal that the VIP and α7 cell models resonant with alpha/theta band input through a dynamic gain analysis.

**SIGNIFICANCE STATEMENT** In the neocortex, ∼25% of neurons are interneurons. Interestingly, only somas of interneurons reside within layer 1 (L1) of the neocortex, but not of excitatory pyramidal cells. L1 interneurons are diverse and believed to be important in the cortical–cortex interactions, especially top-down signaling in the cortical hierarchy. However, the electrophysiological features of L1 interneurons are poorly understood. Here, we systematically studied the electrophysiological features within each L1 interneuron subtype. Furthermore, we build computational models for each subtype and study the mechanisms behind these features. These electrophysiological features within each subtype should be incorporated to elucidate how different L1 interneuron subtypes contribute to communication between cortexes.

## Introduction

Neocortical layer 1 (L1), the most superficial layer of the cerebral cortex, is the main target of extensive “top-down” information conveyed by long-range inputs from other cortical regions and subcortical structures (Cauller, 1995; Garcia-Munoz and Arbuthnott, 2015; D'Souza and Burkhalter, 2017; Schuman et al., 2021). The integration of these inputs into local processing is thought to be mediated by a population of GABAergic inhibitory interneurons (INs) residing in L1 in addition to INs in deeper layers with dendrites or axons in L1 (Larkum, 2013; D'Souza and Burkhalter, 2017; Schuman et al., 2021). To date, modeling studies have mostly neglected L1 INs in computational neuroscience. However, to study the impact of long-range inputs on cortical functions, a detailed understanding of local L1 circuitry is inevitable (Schuman et al., 2021).

To dissect this process, L1 INs have been classified into different subtypes (Schuman et al., 2019). Not limited to the L1, a classification system considering the definitive features that differentiate IN populations, such as gene expression, electrophysiological properties, and morphology, is necessary to study the role of each IN subtype in cortical circuits (Tremblay et al., 2016). Essential findings have been achieved by tagging IN subtypes through genetic strategies (Tremblay et al., 2016), or exploiting the advantages of the Patch-seq technique to gean electrophysiologic and transcriptomic information from the same cell (Gouwens et al., 2020; Scala et al., 2021). In a previous study, we identified four unique populations of INs with somas in L1, each with a distinct molecular profile, morphology, electrophysiology, and connectivity. These four types were canopy cells, neurogliaform cells (NGFCs), cells with high levels of the α7 nicotinic receptor (α7 cells), and vasoactive intestinal peptide (VIP)-expressing cells (Schuman et al., 2019).

However, models *in silico* of the functional properties of these cells are still lacking, which hinders the incorporation of these specialized L1 IN subtypes into circuit models. Models of single neurons have proved to be important for understanding the complex behavior and function of neuronal circuits (Llinás, 1988; Traub and Miles, 1991). From recent data (He et al., 2016; Goff and Goldberg, 2019; Schuman et al., 2019; Prönneke et al., 2020), it is of particular interest to understand the irregular (IR) firing pattern, which among INs is mostly observed in VIP cells (Tremblay et al., 2016). The IR firing was a term initially used to describe the unpredictable firing pattern from trial to trial in response to the same depolarizing current injections, a unique feature observed in cells coexpressing VIP and calretinin from the sensory-motor cortex of rats (Cauli et al., 1997), and a subgroup of cannabinoid receptor-1-positive INs in the somatosensory cortex of mice (Galarreta et al., 2004). Previously, one modeling study suggested the IR firing arose from the noise-introduced transition between bistable states (Stiefel et al., 2013), which required fast activation kinetics of K^+^ channels. In contrast, another study proposed that the clustered spiking depended on the slow inactivation of a Kv1 current (Sciamanna and Wilson, 2011). These hypotheses are worth a reevaluation in our L1 dataset.

In this study, we identify salient electrophysiological features (e-feature) based on the firing patterns within our L1 IN dataset, namely, IR spiking, accelerating (Acc), onset bursting (OB), and spike frequency adaptation (Adap). Further, we systematically examine the distribution of these features across the previously identified subtypes: canopy cells show IR pattern near rheobase; NGFCs are late-spiking, and ACC during current injections; α7 cells display OB; and heterogeneous VIP cells can exhibit OB, Adap, or IR in the same cell. We then construct generic exponential integrate-and-fire (EIF) models for each IN subtype. We especially reproduce IR, OB, and Adap in a generic VIP cell model mimicking a “hero” VIP cell in the dataset. This model includes a slowly inactivating K^+^ channel (SIK), a T-type Ca^2+^ channel, and a spike-triggered Ca^2+^-dependent K^+^ channel. A slow-fast analysis shows that irregularity in VIP cells is square-wave bursting. Furthermore, we compare the heterogeneous firing patterns reproduced by varying parameters and conclude that IR firing is more frequently observed in VIP cells because of the interaction between strong Adap and the SIK channel. We further test the frequency-dependent response of the VIP cell model through a dynamic gain (DG) analysis, and find that the VIP cell model can resonate with alpha/theta band input, enabled by the dynamics of the T-type Ca^2+^ channel.

## Materials and Methods

### Data

The L1 interneuron data used in this study were published previously (Schuman et al., 2019), where a detailed description is available. The data were collected from mice of either gender. The four types of interneurons we used in this study are classified based on a combination of gene markers, electrophysical measurements, morphologic features, and connectivity (for details, see Schuman et al., 2019). Within this dataset, the electrophysiological recordings used extremely small increments of positive current injection (1-10 pA) such that the behaviors around the rheobase have great fidelity. For example, the recording of the “hero” VIP cell used 1 pA as the current step. These fine recordings allow us to study the mechanisms behind different electrophysiological features.

**Figure 1.**
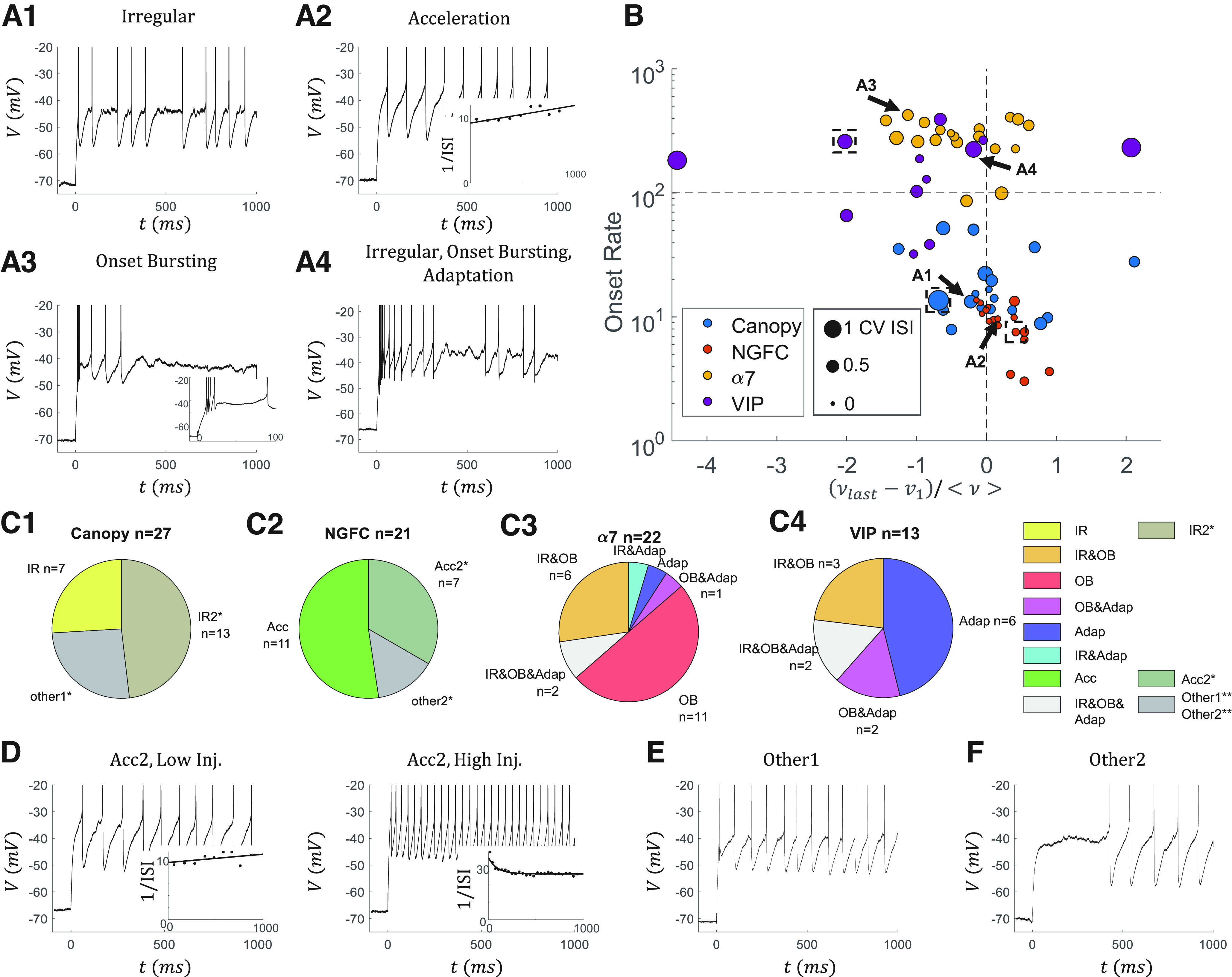
Dominant electrophysiological features of L1 interneuron subtypes. ***A***, Examples of traces that show four different types of features: ***A1***, IR firing, ***A2***, Acc in the firing rate, ***A3***, OB, and ***A4***, Adap in the firing rate. ***A2***, Inset, 1/ISI and the corresponding fitted line. ***A3***, Inset, Zoomed-in traces during the first 100 ms. Traces with ∼10 APs are selected as examples. ***B***, Scatter plot of the L1 INs from four subtypes in an E-feature space (*n* = 69). Each dot represents one IN. Only the cells with a sweep that has ∼9 APs from 100 ms to 1 s are analyzed in this panel (average firing rate <ν> = nAP/0.9 s = 10 Hz). *x* axis shows the normalized change of the instantaneous firing rate (ν = 1/ISI) from the last to the first ISI between 100 ms and 1 s. *y* axis is the onset firing rate, calculated by the first ISI. The size of the point represents the CV ISI (>200 ms) of that sweep. Arrows indicate the cells from ***A***. Dashed boxes represent the “hero” cells to model except the α7 cell (for details, see main text). ***C***, Distribution of the four features in ***C1***, Canopy cells, ***C2***, NGFCs, ***C3***, α7 cells, and ***C4***, VIP cells. *IR2 and Acc2 features are classified by looser criteria than IR and Acc, respectively (see main text). Cells with IR2 or Acc2 are only marked in the canopy cells and NGFCs for simplicity. Eleven of 27 canopy cells, 4 of 21 NGFCs, 9 of 22 α7 cells, and 8 of 13 VIP cells have an IR2 feature. All VIP cells are classified as having an IR or IR2 feature. Five of 27 canopy cells, 8 of 21 NGFCs, 0 of 22 α7 cells, and 0 of 13 VIP cells are classified as having an Acc2 feature. ***D***, Two sweeps from an example cell with an Acc2 feature. The cell shows acceleration in the firing rate when the injection current is low, while Adap when the injection current is high. ***E***, ***F***, **The cells that do not show any feature are indicated as other1 or other2. The other2 cells are late-spiking, but the other1 cells are not.

The cells of which the maximum spike count in all the sweeps is <10 during the 1 s current step is excluded in this study. Only the cells with a sweep that has 6-12 action potentials (APs) from 100 ms to 1 s are analyzed (see Fig. 1*B*). If multiple sweeps from the same cell are available, the sweep with the closest AP numbers to 9 is analyzed.

### AP detection and interspike intervals (ISIs)

The spikes are detected when the voltage trace *V*(*t*) crosses a detection threshold *V_det_*. This threshold is determined as follows: We first set up an upper bound as *V_up_* = min{max(*V*(*t*)), 0 mV}, then a lower bound as *V_low_* = median *V*(*t*)). Next, we set the detection threshold as *V_det_* = max{0.8 *V_up_* + 0.2 *V_low_*, –20 mV}. The ending detection threshold *V_det_* is within the range –20 to 0 mV, and it is robust when the AP maximum voltage and AP threshold vary across different cells. The *i*th AP is detected at *t_i_* if *V*(*t_i_*) < *V_thr_* and *V*(*t_i_* + Δ*t*) ≥ *V_thr_*, with the resolution in our recordings Δ*t* = 0.5 ms. We further exclude any noise-introduced detections within 0.5 ms following another AP.

We calculate the ISI as ISI (*t_i_*) = *t_i_*_+1_ – *t_i_*. We define instantaneous firing (IF) rate as 1/ISI. In some cases, we fit the curves of instantaneous firing rate to an exponential function *a* + *b*exp(–*cx*) by using the MATLAB build-in function *fit*.

### Criteria for electrophysiological features

#### IR

The IR score and CV ISI are calculated for the sweeps with >5 spikes during 500 ms to 1 s. The IR score of a pair of ISIs is defined as 1 – min{ISI(*t_i_*)/ISI(*t_i_*_+1_), ISI(*t_i_*+1)/ISI(ti)}, where *t_i_* > 500 ms. To exclude the potential impact from noise, only the cells with three different ISI pairs, in all the sweeps of the cell, of which the IR score is bigger than the threshold 0.4, are classified as having an IR feature. The IR score of a sweep is defined as the maximum IR score of all ISI pairs of the sweep. CV ISI = std(ISI(*t_i_*))/mean(ISI(*t_i_*)), where *t_i_* > 500 ms. If a cell only has one ISI pair, of which IR score is bigger than the threshold during 200 ms to 1 s, but is not classified as having an IR feature, the cell is classified as having an IR2 feature. In the model, the IR score and CV ISI are measured from 3 to 5 s during the injection period. The IR width is only measured for the cells with an IR feature. It is defined as the number of sweeps with an IR score bigger than the threshold 0.4 times the current step Δ*I_inj_* of the corresponding recordings.

#### Acc

We do a linear regression on the curve of 1/ISI for each sweep to calculate the slope. A cell is classified as having an Acc feature if its slopes from all the sweeps are positive. If a cell has more than two positive slopes (at least two sweeps are Acc) but is not classified as having an Acc feature, the cell is classified as having an Acc2 feature.

#### OB

We fit the corresponding IF curve to an exponential decay function for the sweeps from a given cell with at least four spikes (3 ISIs) during the first 100 ms of the current injection. If all the fitted IF curves drop at least 50% during this 100 ms, and if the first ISI in all these sweeps is lesser than 10 ms, which suggests the onset IF is bigger than 100 Hz for all these sweeps, the cell is classified as having an OB feature.

#### Adap

We fit the IF curves of a given cell during 100 ms to 1 s to an exponential function *f_adap_*(*t*) for the sweeps with more than six spikes (5 ISIs) during this period. The fraction of the dropped firing rate is defined as Adaptation Index (AI = 1 – *f_adap_* (1 s)/*f_adap_* (100 ms)). If the fitted IF curve drops at least 20% (AI > 0.2) in 900 ms for all the sweeps, the cell is classified as having an Adap feature.

### Measurements of electrophysiological properties

The time constant τ of a cell is calculated by fitting the voltage trace from the 100 ms following the end of the sag step to an exponential decay function on each sweep, then averaging across all the sweeps. Resistance *R* is calculated by first dividing the voltage difference (Δ*V*) over the current differences (Δ*I_inj_*) before and during the sag step, then averaging across all the sweeps. Capacity *C* is calculated by dividing the timescale over the resistance in each sweep and then averaging across all the sweeps.

The delay of the first spike is defined as the time of the first spike of the sweep to current injection onset, measured at the sweep with the lowest injection current and at least 2 APs, such that we exclude the possibility that a single spike is triggered by large fluctuation just below the rheobase. We analyze the slope and rheobase of the firing rate curve (f-I curve) by fitting the spike counts during 1 s to a ReLU function *f*(*I*) = *k*max{*I* – *I*_0_, 0}, where the resulting *k* is reported as the f-I slope and *I*_0_ as the rheobase. Only the sweeps with lesser than 40 Hz, or the first five sweeps with APs are used to do the ReLU fitting, such that the fitted curve better represents the behavior around the rheobase.

To measure the properties of APs, we exclude the first two APs, which limits the effect of APs during the OB, and combined remained APs from all the sweeps of which the spike count is <40. For each AP, the maximum voltage is calculated from the 2 ms time window following the trace past the AP detection threshold; then, all the APs are aligned by setting *t* = 0 when the voltage reaches the maximum. Maximum rise and decay slopes are calculated during [–1 ms, 10 ms]. The AP reset is calculated as the minimum voltage during [0 ms, 10 ms]. The AP threshold is calculated as the voltage when the voltage deviation is 20 mV/ms. The halfwidth of AP is calculated as the time of the AP above the midpoint between the maximum voltage and the threshold. Linear interpolation is used in calculating the threshold and halfwidth to improve resolution. Then, all these properties are averaged across all the APs from a cell.

All the measured properties are listed (see Table 2). Some values are not identical as in Schuman et al. (2019) because we exclude cells with <10 spikes.

### EIF models

We implement our models in Python3.9 with the Brian2 package. We use the default ODE solver in Brain2 with a timestep dt = 0.1 ms. Following Fourcaud-Trocmé et al. (2003), our EIF models have the following form:

(1) $$C\frac{dV}{dt}=-((V-V_{L})+\Delta_{T}\exp(\frac{V-V_{T}}{\Delta_{T}}))/R+I_{inj}+I_{\sigma }(t)+<\text{other currents}>,$$

where *V* represents the voltage; *C*, *R* are the passive parameters, capacity, and input resistance; *V_L_* is the reversal potential; *V_T_* is the voltage threshold, and Δ*_T_* is the curvature at *V_T_*; σ is a Gaussian noise term. *I_inj_* is the tonic injection current. *I*_σ_(*t*) = ση(*t*) is the fluctuated synaptic or channel current, where η(*t*) is an Ornstein-Uhlenbeck (OU) process with zero mean and unit variance. For most of the simulation, the correlation time τ_σ_ of the process is τ_σ_ = 0.5 ms, representing an almost-white channel noise. The results are indistinguishable by using a white noise directly. For the simulations in the DG analysis (see Fig. 12), the correlation time τ_σ_ = 0.5 ms, representing the fluctuated synaptic noise.

Noticing that the voltage threshold *V_T_*, representing the voltage where the slope is zero, is not the same as the AP threshold measured from the data, where the slope is 20 mV/ms. All the models have a 2 ms refractory period after each AP.

In addition, we have included different currents to reproduce the rich dynamics of L1 interneurons.

To reproduce the IR and Acc, we include an SIK current as in Sciamanna and Wilson (2011) dynamics:

(2) $$I_{SIK}=-g_{SIK}a^{3}b(V-V_{k}),$$

(3) $$\tau_{a}\frac{da}{dt}=a_{inf}-a=\frac{1}{1+exp(-(V-a_{mid})/a_{sig})}-a,$$

(4) $$\tau_{b}\frac{db}{dt}=b_{inf}-b=\frac{1}{1+exp(-(V-b_{mid})/b_{sig})}-b.$$

where $V_{k}=-90mV,\tau_{a}=6ms,\tau_{b}=150ms,a_{sig}=10mV,b_{sig}=-6mV,a_{mid}=-50mV+V_{\mu },b_{sig}=-65mV+V_{\mu }$. We shifted the mid point of equilibrium curve *a_inf_*, *b_inf_* by *V*_μ_ = 20 mV to have the desired window currents. We vary the conductance *g_SIK_* in different models.

To reproduce OB, we include a T-type Ca^2+^ current as in Smith et al. (2000):

(5) $$I_{T}=-g_{T}m_{T,\infty }h_{T}(V-V_{ca}),$$

(6) $$m_{T,\infty }=\{\begin{array}{ll} 1, & V>V_{h},\\ 0, & V<V_{h}, \end{array}$$

(7) $$\frac{dh}{dt}=\{\begin{array}{ll} -h/\tau_{h}^{-}, & V>V_{h},\\ (1-h)/\tau_{h}^{+}, & V<V_{h}, \end{array}$$

where $V_{ca}=120mV,V_{h}=60mV,\tau_{h}^{-}=5ms,\tau_{h}^{+}=100ms$.

To reproduce Adap, we include a spike-triggered Ca^2+^-dependent K^+^ current, as in Liu and Wang (2001):

(8) $$I_{Adap}=-\gamma C_{ca}(V-V_{k}),$$

(9) $$\tau_{ca}\frac{dC_{ca}}{dt}=\sum_{i}\delta (t-t_{spike}^{\left(i\right)})-C_{ca},$$

where *C_ca_* represents a dimensionless Ca^2+^ concentration in the cell. It jumps by 1 after each spike at $t_{spike}^{\left(i\right)}$.$\tau_{ca}=500ms$.

To tune our model, we first adopt the passive parameters, namely, capacity *C* and input resistance *R*, measured from the data. Next, we take the resting voltage as the clamping voltage in the data, which is ∼–70 mV. Then, we estimate the reset voltage, threshold, and curvature from the phase diagram of the cell. We also estimate the noise term σ based on the SD of voltage below the rheobase. In addition, we consider the change of the gating variable during the APs if an SIK channel is included.

Importantly, we tune the conductance *g_SIK_* of the SIK channel based on the IR score, CV ISI, acceleration, and delay of the first spike; we modify the conductance *g_T_* of T-type Ca^2+^ based on the firing rate during OB, and we choose the strength of Adap γ based on the AI curve.

We further adjust all the parameters in the model to reproduce all features the best.

All parameter values of the model are listed in Table 1.

**Table 1.** Parameters used in the models*^a^*

	Canopy	NGFC	α7	VIP
Resistance R (mΩ)	86	163	120	400
Capacity C (pF)	80	80	80	80
Reversal potential *V_L_* (pF)	–67	–70	–70	–69
Threshold *V_T_* (pF)	–38	–40	–39	–40
Reset voltage *V_T_* (pF)	–52	–52	–50	–50
Curvature Δ*_T_* (mV)	1	1	0.5	1
Noise std σ (pA)	20	8	10	10
Conductance *g_SIK_* (nS)	120	30	—	150
Jump of slow gating variable Δ*b*	–0.002	–0.004	—	–0.001
Conductance *g_T_* (nS)	—	—	8	10
Adap strength γ (nS)	—	—	—	0.1
Rheobase (pA)	366.2	177.5	244.3	177.7
f-I slope (Hz/pA)	1.64	0.933	1.28	0.33

*^a^*The rheobase and f-I slope are measured by fitting the curve of firing-rate at the steady state to a ReLU function.

### Dynamic gain

The DG function *DG*(*f*) is defined as the ratio of the response and the input at a specific frequency. We followed the exact method as described by Ilin et al. (2013). Here, the response firing rate function is defined as a summation of δ functions at spike time *t_i_*:

(10) $$\nu (t)=\sum_{i=1}^{n}\delta (t-t_{i})$$

As proposed by Higgs and Spain (2009), we calculated the DG function in the Fourier space as follows:

(11) $$DG(f)=\left|\frac{ℱ(\nu (t))}{ℱ(I_{\sigma }(t))}\right|=\left|\frac{ℱ(C_{I\nu })}{ℱ(C_{II})}\right|.$$

The numerator is the Fourier transformation of input–output correlation. Since the firing rate ν(*t*) is a sum of δ functions, *C*_*I*ν_ can be simplified into the averaging current in the time windows around each spike as follows:

(12) $$F(C_{I\nu })=<\nu >\cdot F(STA)$$

where *STA* represents the spike-triggered average current. We use a 1 s window and frequency-dependent Gaussian filters to suppress noise.

Since the *I*_σ_(*t*) is a standard OU process, the denominator can be calculated theoretically (see also Zhang et al., 2022), as follows:

(13) $$F(C_{II})=\frac{2\tau_{\sigma }\sigma^{2}}{1+{\left(2\pi \tau_{\sigma }f\right)}^{2}}$$

At last, the DG is normalized by the value at 1 Hz *DG*(1).

The 5% confidence threshold is calculated by bootstrapping as in Borda Bossana et al. (2020). We first generated 500 surrogated response ν*_i_*(*t*) trials by shuffling the original ISIs. Then we calculated the DG functions *DG_i_*(*f*) of these surrogated responses. The confidence curve is calculated based on each frequency's mean and variation from the *DG_i_*(*f*).

To calculate the high-frequency profile, we follow Borda Bossana et al. (2020). The cutoff *f*_0_ is defined as the DG dropps to 70% *DG*(1). To describe the exponential decay of DG at large frequencies, we fit the *DG*(*f*) between *DG*(*f*_0_) and 0.2 *DG*(*f*_0_) to *y* = *F*(*f*) = *bf*^–α^, where α represents the decay constant.

### Experimental design and statistical analysis

We analyzed the original pClamp data in MATLAB (The MathWorks) using a self-developed package. The models in this study were developed in Python 3.9.7 (Python Software Foundation) with Brian2 package (Goodman and Brette, 2008). All statistical analysis was performed in MATLAB. The developed toolbox and the models can be accessed through ModelDB: http://modeldb.yale.edu/267682.

The full dataset is available on request.

## Results

### Identifying dominant electrophysiological features of L1 interneuron subtypes

Previously, we separated the L1 IN population into canopy cells, NGFCs, α7 cells, and VIP cells based on a combination of genetic markers, morphology, connectivity, and electrophysiological features, such as input resistance, sag, and latency to first spike (Schuman et al., 2019).

Here, we systematically studied electrophysiological features of firing patterns based on the analysis of all the sweeps in our dataset. We first identified four features of interest in this L1 dataset (Fig. 1*A*). Following the Petilla terminology (Ascoli et al., 2008), these features are IR firing behavior, acceleration of the firing rate (Acc), OB, and Adap of the firing rate. To show how salient these features exist in our dataset, we plot each cell in an e-feature space (Fig. 1*B*). To compare across cells, we used the sweep where the firing rate is ∼10 Hz during the 100 ms to 1 s of the current step. The IR is represented by the CV ISI (size), the Acc and Adap features are represented by the normalized change of the firing rate (*x* axis), and the OB is characterized by the onset firing rate (*y* axis). Although this is a useful tool, it is limited in two ways. First, when the cell fires irregularly, the change in the firing rate may reflect the irregularity but not the trend in the firing rate. Second, the information that resides outside the 10 Hz sweeps is lost in this analysis. Still, different IN subtypes (colors) are separated in this space. Canopy cells show IR in many cells but no other features; NGFCs only show Acc; α7 cells are OB without exceptions; and VIP cells are heterogeneous but mostly show IR and Adap. The traces in Figure 1*A* are marked by arrows in Figure 1*B*.

Next, we set quantitative criteria based on all the sweeps, but not just one sweep, to better determine if each cell shows these four features (Fig. 1*C*). Here, we include more cells than in Figure 1*B*, since having a sweep of 10 *Hz* is not required. Traditionally, irregularity is used to describe a unique, unpredictable firing pattern (Galarreta et al., 2004). This can be further reflected in the abrupt change of the ISI (Fig. 1*A1*). To quantify it, we define an IR score of a pair of neighboring ISIs as 1 minus the ratio of the smaller ISI and the larger ISI. If one cell has at least three pairs of ISIs during the last 500 ms of the depolarizing pulse that have an IR score bigger than the threshold of 0.4, we consider the cell as having an IR feature. We find the IR score is more robust than the traditional measurement CV ISI in quantifying the IR firing for the following reasons. On the one hand, we have fine recordings around the rheobase, such that we can collect ISI pairs from different sweeps to distinguish the IR firing from pure noise. On the other hand, many L1 INs show Acc or Adap, which is hard to distinguish from IR by CV ISI.

We only use the last 500 ms to avoid the potential false-positive cases because cells with strong Adap or OB can change their firing rate rapidly at the onset of the current injection period. We also define a looser criterion for having an IR2 feature, that is, if a cell does not display an IR feature but has at least one pair of ISIs during the latter 800 ms. If a cell with an IR2 feature may have an IR feature if the recordings were done with finer current steps, but it may not if the ISI pairs with large IR scores are purely introduced by noise. IR is widely observed in L1 INs, except for NGFCs (Fig. 1*B*,*C*). Noticeably, all the VIP cells have an IR or IR2 feature.

We consider a cell as having an Acc feature if it shows acceleration in the firing rate in all the sweeps. A looser criterion, which only requires one cell to have at least two sweeps around rheobase to show the acceleration in the firing rate, is applied to consider a cell as having an Acc2 feature. These cells with an Acc2 feature usually show acceleration when the injection current is low, but Adap when the injection current is high (Fig. 1*D*). Cells with an Acc feature are uniquely observed in NGFCs. Further, most NGFCs are considered as having an Acc or Acc2 feature (Fig. 1*C2*), and some canopy cells are found to have an Acc2 feature (5 OF 27).

Then, to distinguish OB and Adap, we define OB and Adap based on different time frames during the current step. We consider a cell to have an OB feature if, first, the onset firing rate is >100 Hz (first ISI is lesser than 10 ms) in all the sweeps with at least five APs; and second, the firing rate drops at least 50% in the first 100 ms in all the sweeps. We consider a cell to have an Adap feature if the firing rate drops at least 20% from 100 ms to 1 s in all sweeps. The OB feature is observed in most of the α7 cells (20 of 22, the two α7 cells without OB features are just below the OB cutoff in Fig. 1*B*), and some of the VIP cells, while Adap is observed in most of the VIP cells and some of the α7 cells (5 of 22, Fig. 1*C3*,*C4*).

**Figure 2.**
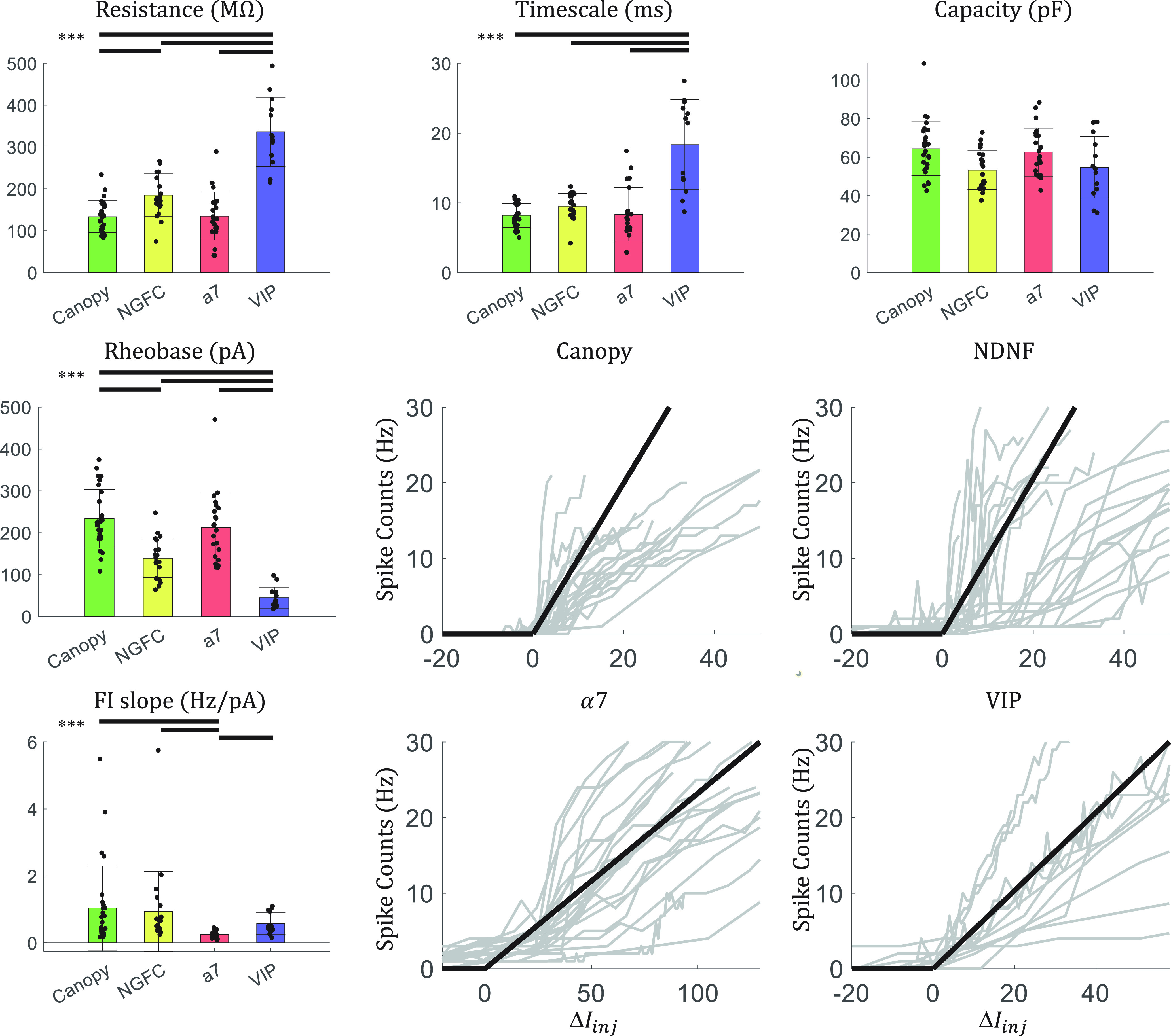
Electrophysiological features of the L1 interneurons. Bars above suggest the corresponding pair is significantly different (*p* < 0.001, Mann–Whitney *U* test). Mean ± SD. Scatters shows the values measured from individual cells. Jitters are added for better visualization. Rheobase and FI slope are measured by fitting the spike-count curve to an ReLU function. Lines are aligned by the rheobase. Bold black lines indicate the ReLU function with the mean FI slope. Gray lines indicate individual spike-count curves of individual cells.

The cells with no associated features are marked with other1 or other2. While the other1 canopy cells show no signature (Fig. 1*D*), the other2 NGFCs are all late-spiking (Fig. 1*E*). The passive parameters are listed in Table 2 and shown in Figure 2. The detailed characterizations of individual cells are listed in Extended Data Table 1-1.

**Table 2.** Intrinsic electrophysiological properties of the four L1 IN subtypes*^a^*

	Canopy (*n* = 27)	NGFC (*n* = 21)	α7 (*n* = 22)	VIP (*n* = 13)
Timescale (ms)	8.23 ± 1.72	9.53 ± 1.84	8.37 ± 3.86	18.34 ± 6.45
Resistance (mΩ)	133.5 ± 38.2	185.4 ± 50.4	135.3 ± 57.3	336.6 ± 83.0
Capacity (pF)	64.4 ± 14.0	53.3 ± 10.1	62.6 ± 12.5	45.1 ± 16.0
Delay (ms)	29.3 ± 15.1	497.9 ± 249.9	37.4 ± 17.4	113.8 ± 42.7
Rheobase (pA)	233.9 ± 70.1	139.10 ± 46.2	212.6 ± 82.1	25.0 ± 25.6
fI slope (Hz/pA)	1.04 ± 1.26	0.94 ± 1.19	0.25 ± 0.11	0.58 ± 0.32
IR width*^b^* (pA)	17.9 ± 25.3	—	91.8 ± 104.2	99.0 ± 121.9
AP rise (mV/ms)	365.3 ± 83.8	419.7 ± 78.2	398.3 ± 99.9	480.1 ± 221.1
AP decay (mV/ms)	−98.0 ± 23.9	−88.5 ± 19.8	−99.5 ± 29.3	−185.9 ± 67.7
AP halfwidth (ms)	0.610 ± 0.107	0.657 ± 0.158	0.659 ± 0.189	0.456 ± 0.151
AP reset (mV)	50.0 ± 3.6	−52.9 ± 2.5	−47.8 ± 3.2	−52.8 ± 6.6
AP threshold (mV)	−34.9 ± 3.0	−32.4 ± 1.7	−33.8 ± 2.3	−35.2 ± 4.9
AP max Volt (mV)	32.0 ± 6.4	35.6 ± 5.0	34.8 ± 5.8	38.9 ± 11.5

*^a^*Data are mean ± SEM.

*^b^*The IR width is only measured for the cells with an IR feature within each subtype (*n* = 7, 9, and 5 for canopy, α7, and VIP cells, respectively) (for details, see Materials and Methods). The values are not exactly the same as the previous study (Schuman et al., 2019) because we exclude the cells with <10 APs in the sweep with the maximum injection current.

10.1523/JNEUROSCI.1876-22.2023.tab1-1 Table 1-1 The analysis on individual L1 INs. Download Table 1-1, XLSX file.

In this study, we want to have parsimonious models that reproduce the spectrum of these behaviors and help understand the mechanisms behind these behaviors. To achieve these, instead of tuning our model based on the averaged features of a subtype, we choose one “hero” cell from each subtype that only shows one type of the electrophysiological feature of interest and reproduce the behavior of that cell. However, readers should be aware that our model does not consider heterogeneity within each cell type. The analysis on individual cells is provided in Extended Data Table 1-1 if the heterogeneity itself is of interest. Canopy, NGFC, and VIP “hero” cells are marked by the dashed boxes in Figure 1*B*. The closest sweep of α7 “hero” cell has 17 APs after 100 ms, with CV ISI = 0.09, *d*ν /<ν> = 0.18, and onset firing rate = 235.3 Hz.

We do this because the averaged “cell” may not be physically plausible because of the large heterogeneity within an interneuron subtype. We choose to make individual neuron models based on the EIF neuron (Fourcaud-Trocmé et al., 2003), but not Hodgkin-Huxley models, to minimize the number of parameters we need to tune. In the following sections, we first reproduce IR in a canopy cell model; second, capture the Acc and delayed spiking in an NGFC model; and third, generate the OB in an α7 cell model. Finally, we model a VIP cell that shows IR, OB, and Adap simultaneously.

### IR firing is reproduced in a canopy cell model with an SIK channel

**Figure 3.**
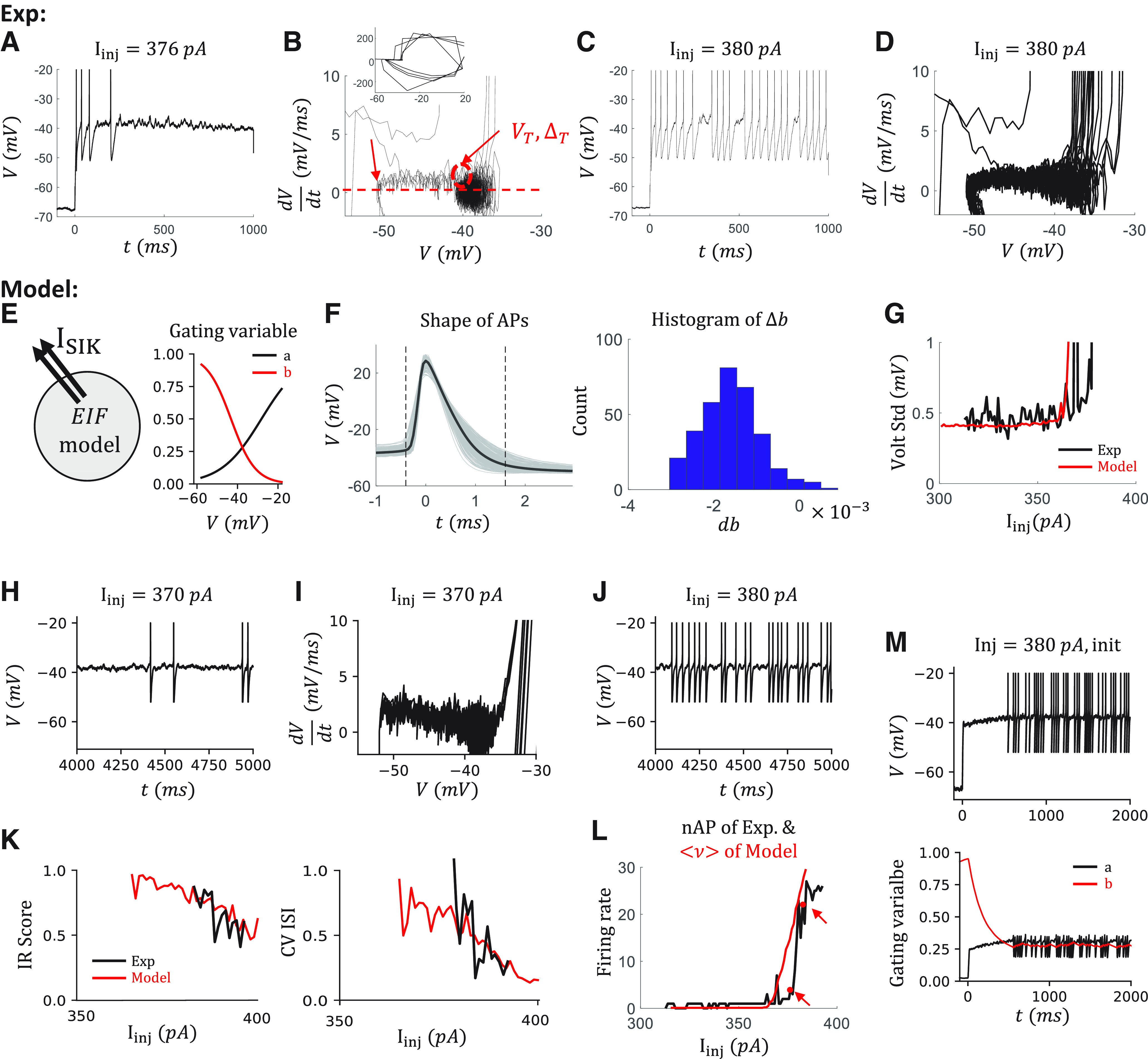
Modeling IR spiking of a canopy cell with an SIK channel. ***A***, The voltage trace of an example canopy cell at I_inj_ = 376 pA. ***B***, Phase diagram of the cell. Inset, The corresponding zoom-out diagram. *y* axis is the slope of the voltage trace, which is proportional to the net current to the cell. Red arrows indicate where we estimate the value of reset, curvature, and firing threshold of the corresponding model. ***C***, ***D***, The trace and the phase diagram at I_inj_ = 380 pA. ***E***, The sketch of the canopy cell model (left) and the equilibrium value of the gating variables of the SIK channel (right). ***F***, Δb estimation. Left, The shapes of all the APs from all the sweeps of the same cell. We exclude the first two APs in every sweep. APs are aligned at the maximum of the voltage. Dashed lines indicate *t* = –0.4, 1.6 ms, respectively. Voltage traces of these 2 ms time windows are used to calculate the jump of the slow inactivation variable Δb. Right, The histogram of Δb with an initial value *b_init_* = 0.28. The value *b_init_* = 0.28 is the average value after 3 s of injection in the simulation. The average change is <Δb> = –0.0020. ***G***, The voltage SD measured between 500 ms and 1 s during the current injection from the data (black) and the model (red). ***H***, ***I***, The voltage trace and the phase diagram of the canopy cell model at I_inj_ = 370 pA. ***J***, The voltage trace at I_inj_ = 380 pA. ***K***, The comparison of irregularity from the data and the model by IR score (left) and by CV ISI (right). ***L***, The comparison of firing rate from the data, estimated by the number of APs during the 1 s current injection, and from the model, estimated by the steady-state firing rate. Red arrows indicate *I_inj_* = 376, 380 *pA*. ***M***, The initial dynamics are not considered in this model. Top, The voltage trace that includes the initial transient part when I_inj_ = 380 pA. Bottom, The transient dynamics of gating variables of the SIK channel. Black and red lines indicate the fast and slow variables *a* and *b*, respectively.

Since canopy cells with an IR feature do not have any other of the features under consideration, modeling IR on canopy cells does not need to consider the potential interactions between different features (Fig. 1*C1*). From the selected “hero” canopy cell, we observed substantial subthreshold fluctuations ∼–40 mV from the voltage traces just above the rheobase (Fig. 3*A*,*C*). This is more obvious in the phase diagram of the cell (Fig. 3*B*,*D*), where we plot the deviation of the voltage, which is proportional to the net current into the cell, over the voltage. A quasi-stable point is observed ∼–40 mV where the net current goes <0, suggesting some outward current is activated during this range.

To mimic the IR at the latter 500 ms during the current injection, we include an SIK channel in an EIF model (Fig. 3*E*, left). The major parameters of an EIF model, namely, reset voltage, firing threshold, and curvature at the firing threshold, can be estimated from the phase diagram directly (Fig. 3*B*, red arrows). The slow-inactivation gating variable from the SIK channel can introduce the clustered spiking, which has been known (Wang, 1993). Here, the model of the SIK channel follows the model of a Kv1 channel in PV^+^ interneurons (Sciamanna and Wilson, 2011), which contains one fast activating gating variable *a* and one slow inactivating gating variable *b*. We shift the equilibrium value of the gating variables to a more depolarized value such that the window current of this channel is ∼–40 mV (Fig. 3*E*, right). As argued by Sciamanna and Wilson (2011, their Fig. 8), this is plausible by taking into account that the Kv1 channels are localized on the axon initial segment but not on the soma.

To tune the model, we further consider the change of the gating variable during the APs. We can estimate the change of the slow variable Δ*b* after each AP by solving the differential equation of *b* while replacing the voltage term by the shape of APs (Fig. 3*F*). The initial value of *b* is chosen based on our simulation. This is plausible because the dynamics of *b* are much slower (time constant τ_b_ = 150 ms) than the time window of APs (refractory period of the model 2 ms). Choosing different Δ*b* in the model has little impact on the simulation results. Since the jump of the fast variable Δ*a* does not change the dynamics of the model because of the rapid convergence to the equilibrium point (τ_a_ = 6 ms, Fig. 3*M*), we set Δ*a* = 0. Also, we estimate the noise term σ based on the SD of voltage before or around the rheobase current during 500 ms to 1 s of the depolarization current step (Fig. 3*G*). Importantly, we tune the conductance *g_SIK_* of the SIK channel based on the IR score curve. At last, we adjust all the parameters in the model to best reproduce other cell features, namely, the rheobase, the slope of the f-I curve, and the firing behavior of individual sweeps.

The resulting model can reproduce the IR behavior observed in the cell (Fig. 3*H–J*) and the changing of IR behavior when the injection current varies (Fig. 3*K*). The firing rate is also comparable between the data and the model (Fig. 3*L*).

However, since we focus on the irregularity in this section, the dynamics around the beginning of the current injection are not modeled (Fig. 3*M*). As a result, the model shows a delayed-firing feature (Fig. 3*M*, top), because of the slow inactivation of SIK channel (Fig. 3*M*, bottom).

### Mechanism of IR firing in the canopy cell model

**Figure 4.**
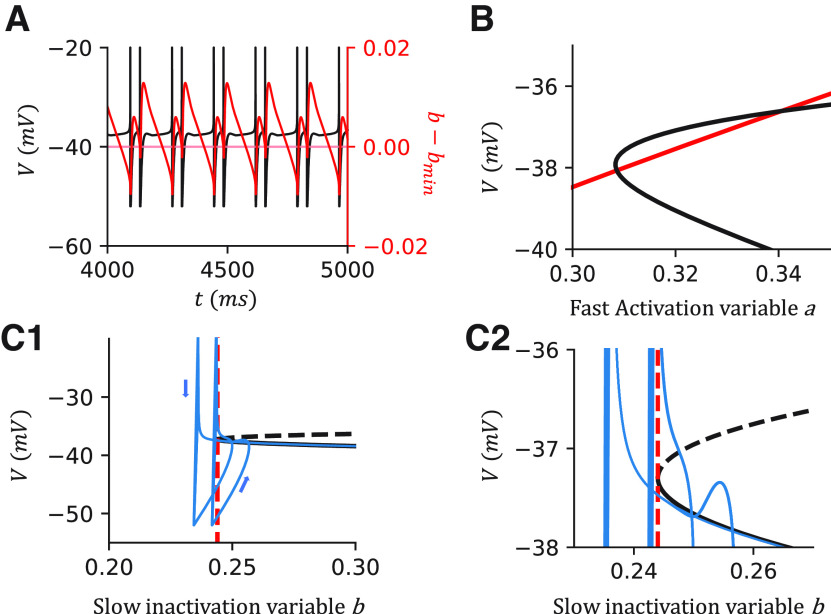
A fast-slow analysis of the canopy cell model shows that the irregularity results from the square-wave bursting. ***A***, Sample simulation when I_inj_ = 375 pA without noise. Black line indicates the voltage trace. Red line indicates the difference between *b* and *b_min_*. Red dashed line is at *b* – *b_min_* = 0, suggesting the boundary between the resting state and the firing state of the cell. ***B***, Nullclines when I_inj_ = 375 pA, *b* = 0.27. The intersections indicate the fixpoints of the fast manifold. ***C***, The bifurcation diagram when varying *b* with I_inj_ = 375 pA. The system undergoes an SNIC. Black solid line indicates the stable branch. Dashed line indicates the unstable branch. Blue line indicates the simulation of the canopy cell model while we remove the noise term σ = 0. Red dashed line indicates the minimum *b* that the fast manifold has a global SS. Arrows indicate the direction of the dynamics. ***C1***, Dynamics of the entire AP range. ***C2***, Zoomed-in dynamics around the bifurcation point.

In our model, The IR feature is reproduced by including an SIK channel. To understand the mechanism, we did a fast-slow analysis on our model (Fig. 4). Excluding the noise term by setting σ = 0, we observe clustered spiking in the model (Fig. 4*A*). We further assume the slow gating variable *b* is a constant and analyze the dynamics of the fast manifold defined by the voltage *v* and the fast activation variable *a*. As expected for the EIF model, the system has one stable state (SS) and one unstable steady state (US) when the injection current is low or the outward current is large (Fig. 4*B*). The SS branch and US branch emerge through a saddle-node bifurcation on an invariance cycle (SNIC) by increasing *b* over the bifurcation point *b_min_* (Fig. 4*C*). In the simulation of the full system (Fig. 4*A*,*C*), the slow variable *b* changes across the bifurcation point *b_min_* over time. Thus, the full system oscillates between a resting state (the global SS exists) and a spiking state (the global SS vanishes). This dynamic is known as square-wave bursting (Rinzel, 1987; Wang and Rinzel, 1995; Rinzel and Ermentrout, 1998). Adding noise to the system σ > 0, the oscillation between the resting and spiking states mimics the IR firing patterns.

### Spike frequency acceleration and delayed firing are reproduced in an NGFC model

**Figure 5.**
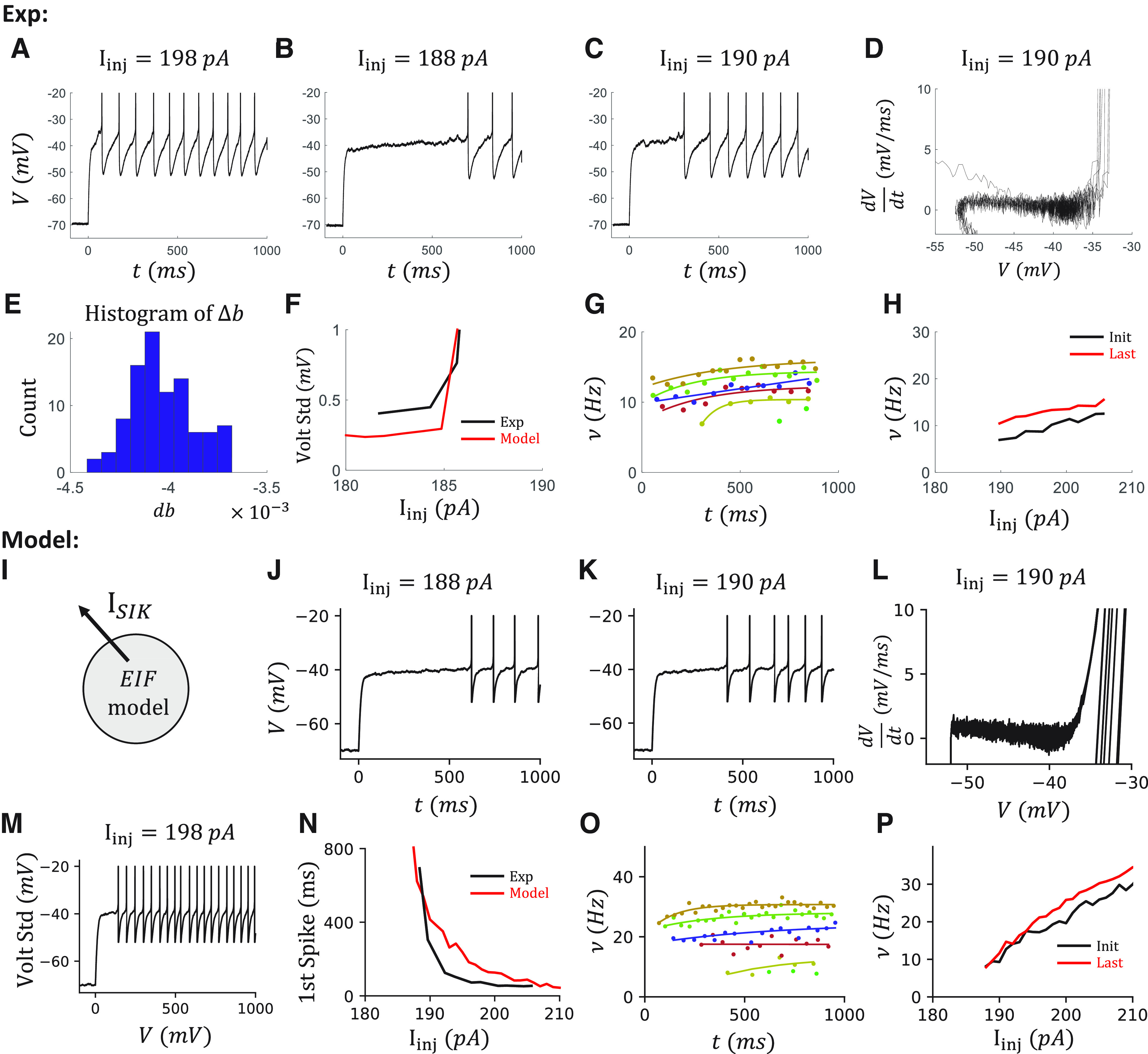
Modeling the acceleration and late-spiking of an NGFC with a smaller SIK conductance. ***A-C***, Voltage traces of the selected NGFC at I_inj_ = 198, 188, 190 pA. ***D***, The phase diagram of the cell at I_inj_ = 190 pA. ***E***, The histogram of Δb with an initial value *b_init_* = 0.4. The average change is <Δb> = –0.0040. ***F***, The voltage SDs of the data and the model. ***G***, The instantaneous firing rate ν = 1/ISI over time. We fit the ISIs (dot) to an exponential decay curve (line) for different injection currents I_inj_ = 188, 190, 194, 198, 202, 206 pA (color). ***H***, The initial firing rate (black) and the last (red) firing rate at different injection currents. ***I***, The sketch of the NGFC model. ***J-M***, Model results correspond to ***B-D***, ***A***. ***N***, The time of the first spike decreases fast when increasing the injection current in both the data and the model. ***O***, ***P***, The instantaneous firing rate ν increases over time in the model. Organized as in ***G***, ***H***.

After capturing the dynamics of IR, we next model the acceleration observed in an NGFC. As shown in Figure 5, the Acc is accompanied by the late onset of spiking (Fig. 5*B*,*C*). Similarly, as in tuning a canopy cell model, we further characterize the cell based on the corresponding phase diagram (Fig. 5*D*) where a similar quasi-stable point is observed at ∼–40 mV, the jump of change of the slow variable Δ*b* after each AP (Fig. 5*E*), and the noise level (Fig. 5*F*). Importantly, the firing rate slowly accelerates over the 1 s injection period (Fig. 5*G*,*H*).

Since the delayed firing is associated with the SIK current (Storm, 1988), along with our observations, we tune an EIF model with the same SIK channel that has the identical dynamics as in the canopy cell model to reproduce the ACC feature (Fig. 5*I*). Compared with the canopy cell model, we reduce the SIK conductance *g_SIK_* to avoid the IR. The relationship between SIK conductance *g_SIK_* and the IR will be discussed later in Figure 11. After a similar tuning procedure, the resulting model is capable of reproducing the Acc feature observed in an NGFC (Fig. 5*J–P*). In addition, the time of the first AP drops fast when it increases the injection current, and our model is capable of reproducing this behavior (Fig. 5*N*).

**Figure 6.**
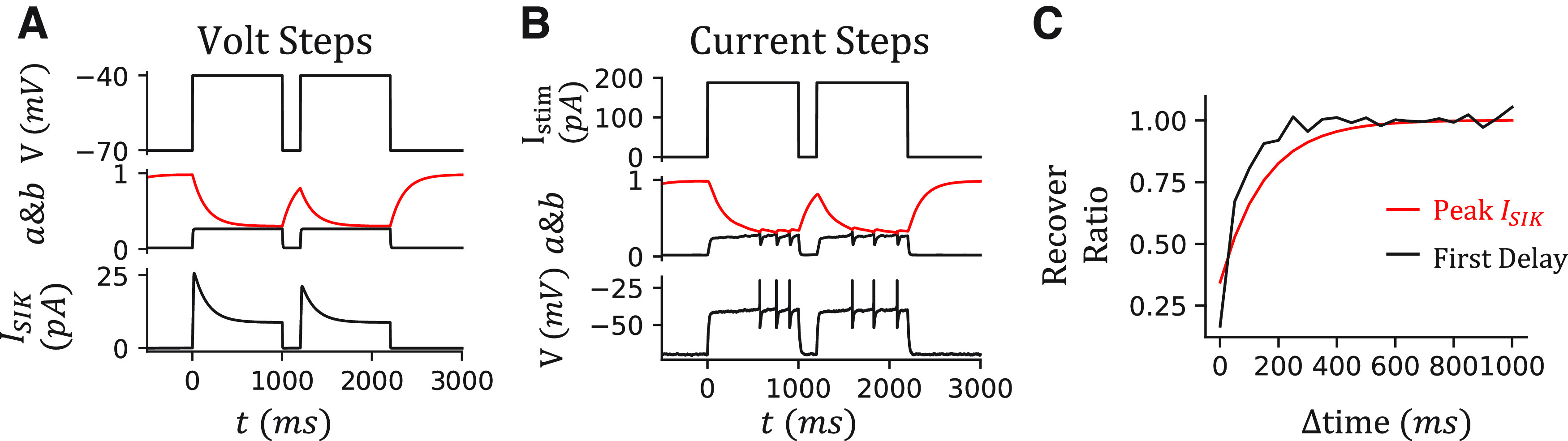
Recovering dynamics of the NGFC model. ***A***, Simulation of the two voltage steps V_clamp_ = –40 mV with a resting time Δtime = 200 ms. From top to bottom, the voltage of the model, gating variables *a* (black) and *b* (red), SIK current *I_SIK_*. ***B***, Simulation of the two current steps I_inj_ = 188 pA with a resting time Δtime = 200 ms. From top to bottom, injection current, gating variables *a* (black) and *b* (red), the voltage of the model. ***C***, The peak SIK current and delay of the first spike recover with a long resting time Δtime. The recover ratio of the peak SIK current (red) is calculated by comparing the maximum *I_SIK_* during the second voltage step to that during the first voltage step. The delay of the first spike is calculated from the current steps (black).

The delayed firing and the acceleration come from the slow inactivation gating variable *b* of the SIK current. To directly show that, we simulate the model with two sequential voltage steps and current steps with a variable resting time Δtime (Fig. 6*A*,*B*). During the first voltage step (Fig. 6*A*), the model neuron is clamped at –40 mV. The fast-activating gating variable *a* opens first and increases the SIK current. Then, the slow-inactivating variable *b* gradually closes and decreases the SIK current. The difference between the onset and steady SIK current is ∼15 pA in our NGFC model. During a rheobase current step (Fig. 6*B*), the model neuron depolarizes to a subthreshold voltage but is held there by the fast-activating SIK current. It is only released to fire when the SIK current inactivates enough. During a current step that is above the rheobase, the SIK current also inactivates gradually, which leads to an acceleration of the firing rate. Further, the strength of the SIK current is subjected to the initial state of the slow variable *b*. If the second voltage step or current step is close to the first, the slow variable *b* does not fully restore to the open state. As a result, the peak of the SIK current and the delay of the first spike is smaller in the second step compared with the first step (Fig. 6*C*).

### OB is reproduced in an α7 cell model

**Figure 7.**
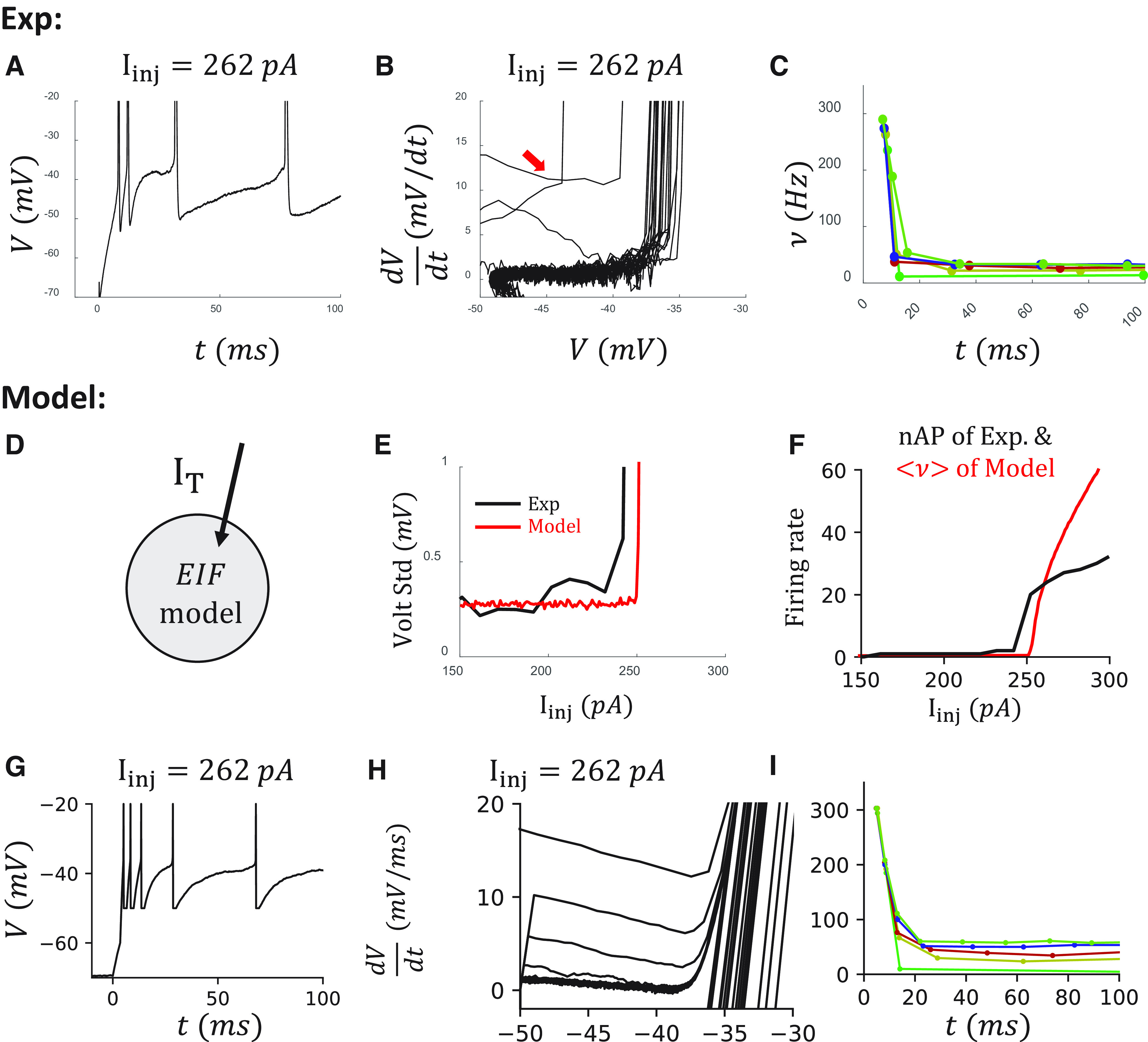
Modeling the OB of an α7 cell with a T-type calcium channel. ***A***, The voltage trace at I_inj_ = 262 pA. ***B***, Phase diagram of the cell. Red arrow indicates the trajectory from the first AP within the OB period. ***C***, Instantaneous firing rate shows a sudden drop in the first 40 ms of the injection period. Colors represent sweeps with different injection currents, I_inj_ = 252, 262, 272, 282, 292 pA. ***D***, The sketch of the α7 cell model. ***E***, The voltage SDs of the data and the model. ***F***, The firing rates from the data and the model. ***G-I***, Modeling results organized as in ***A-C***.

We then studied the OB behavior, which is observed in most of the α7 cells. Cells with an OB feature show a characteristic high onset firing rate and a fast dropping of the firing rate during the first 40 ms (Fig. 7*A*). In the phase diagram of the cell, several lines are above the spiking cycle (Fig. 7*B*, red arrow), which indicates the existence of a transient current that is less dependent on the voltage. Further, the onset firing rate is ∼270 Hz across sweeps with different injection currents. The firing rate robustly drops below 50 Hz within 40 ms (Fig. 7*C*).

As previously suggested (Schuman et al., 2019) and demonstrated (Schuman et al., 2021), OB in α7 cells is dependent on the expression of a T-type Ca^2+^ channel. This was also demonstrated in bursting L2/3 VIP cells (Prönneke et al., 2020). We thus include a T-type Ca^2+^ current in our EIF model for an α7 cell model (Fig. 7*D*) based on the reported dynamics of T-type Ca^2+^ channels from a thalamocortical relay neuron model (Smith et al., 2000). After tuning the T-type Ca^2+^ channel conductance *g_T_* to the OB in the data, along with noise level σ (Fig. 7*E*) and other passive parameters, we can reproduce the high onset firing rate and the fast dropping of the firing rate during the first 40 ms in the α7 cell model (Fig. 7*F–I*).

### IR, OB, and adap are simultaneously reproduced in a VIP cell model

**Figure 8.**
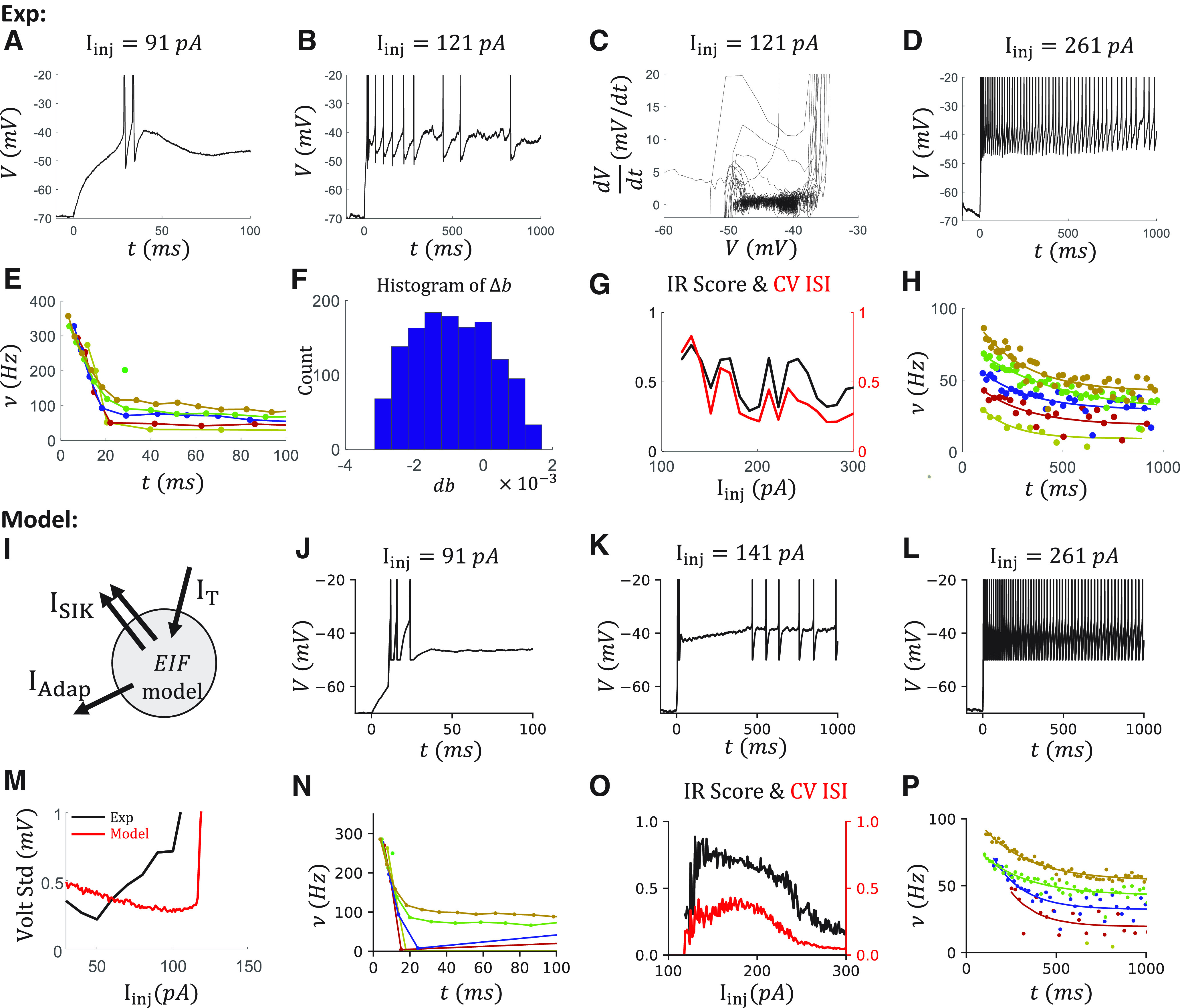
Reproducing the OB, IR firing patterns, and adaption simultaneously in a VIP cell model. ***A-D***, Voltage traces at I_inj_ = 91, 121, 261 pA, which are examples to show OB, IR, and Adap, respectively. ***E***, The instantaneous firing rates ν from 0 to 100 ms that show the OB. Colors represent sweeps with different injection currents I_inj_ = 91, 131, 171, 212, 251, 291 pA. ***F***, The histogram of Δb with an initial value *b_init_* = 0.25. The average change is <Δb> = –0.0009. ***G***, The ISI ratio (black) and CV ISI (red) curves that show the irregularity. ***H***, The instantaneous firing rates ν between 100 ms and 1 s that show the Adap. Color coding is the same as in ***D***. ***I***, Sketch of the VIP cell model. ***J-P***, Corresponding modeling results. ***M***, SD of the voltage of the data and the model.

After reproducing the IR, Acc, and OB features individually, we turn to the complex dynamics observed in VIP cells. Most VIP cells show more than one considered feature (Fig. 1*B4*). For example, OB (Fig. 8*A*,*E*), irregularity (Fig. 8*B*,*G*), and Adap (Fig. 8*D*,*H*) are observed in the same selected VIP cell. All these features can be simultaneously reproduced with an EIF model with a T-type Ca^2+^ channel, an SIK channel, and a spike-triggered small conductance K^+^ channel (Fig. 8*I*).

As described in the modeling of an α7 cell (Fig. 7), the OB has the characteristic high onset firing rate and a fast dropping in the firing rate during the first 40 ms. This was reproduced by tuning the conductance *g_T_* of the T-type Ca^2+^ channel, as in the α7 model.

In addition, the IR score is highest in the sweeps around rheobase, and it decays slowly with increasing injection current (Fig. 8*G*). The IR score remains >0.4, which is the threshold to detect IR, even in a 1 s sweep with 60 APs with injection current *I_inj_* = 300 pA. Above, we reproduced IR spiking by including a strong SIK current in the canopy cell model. However, the IR score drops fast in the canopy cell model when increasing the injection current (Fig. 3*K*).

**Figure 9.**
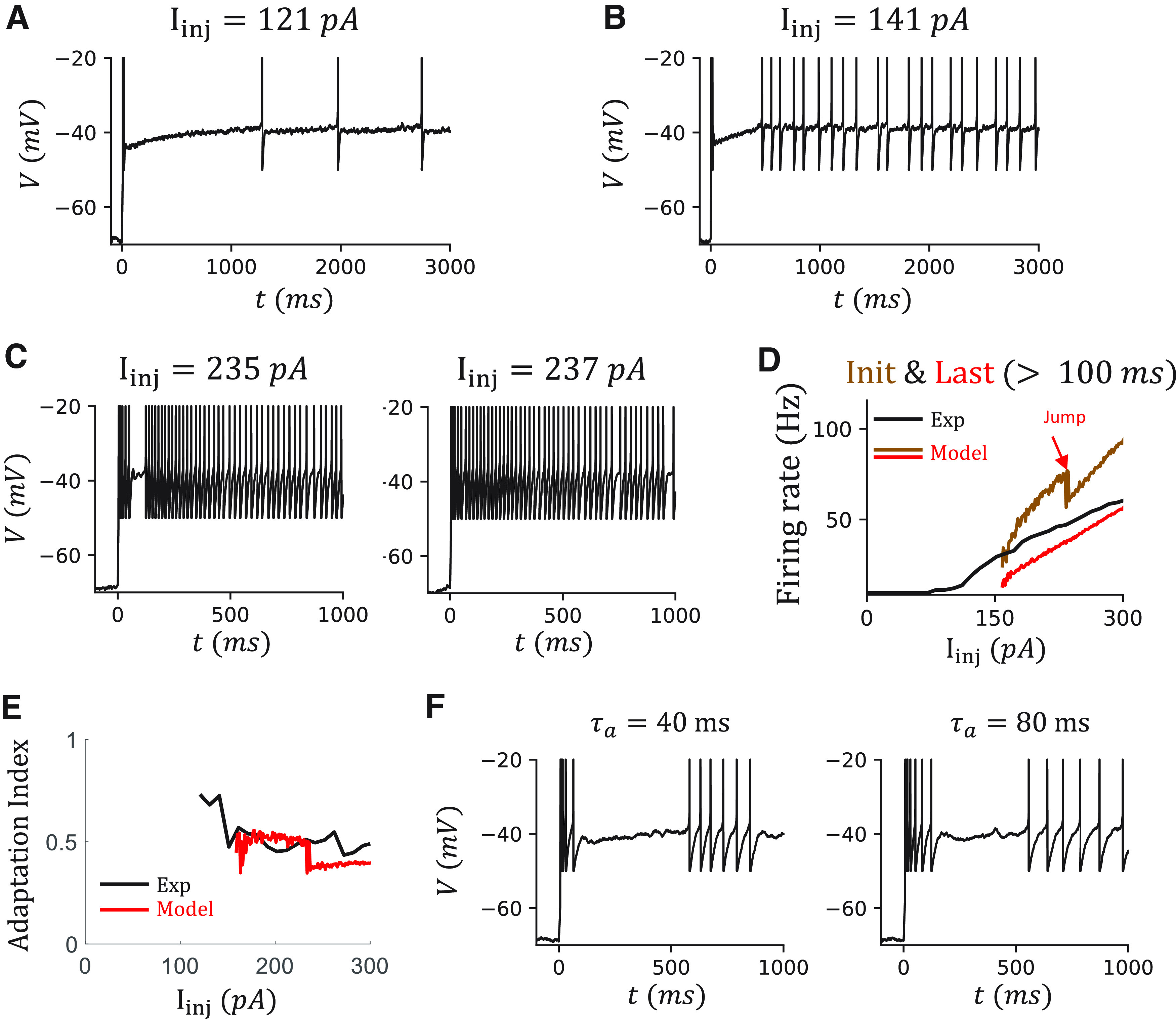
More results of the VIP cell model. ***A***, ***B***, The voltage traces of the model in 3 s at I_inj_ = 121, 141 pA. ***C***, The voltage traces of the model at I_inj_ = 235, 237 pA. The jump in the curve of the initial firing rate is because of the diminishing of the silent period after the OB. ***D***, The comparison of the firing rates ν of the data and the model. The ν of the model is measured as the number of APs during 1 s current injection. The initial and last (steady-state) firing rates ν of the model are measured from 100 to 1500 ms. ***E***, The AI from the data (black) and the model (red), measured based on the fitting of 1/ISI from 100 ms to 1 s. ***F***, Voltage traces when varying the time constant of fast activation variable τ_a_ = 40, 80 ms with an injection current I_inj_ = 141 pA. The firing pattern around 100 ms is more comparable between the data and the model. However, τ_a_ is not biologically plausible anymore (original τ_a_ = 4 ms).

Further, strong Adap is also observed in the VIP cell in the last 900 ms (Fig. 8*D*), where the firing rate drops exponentially to a saturation value (Fig. 8*H*). To quantify that, we define the AI as the fraction of the firing rate that drops during this 900 ms period. For this VIP cell, AI is ∼0.5 in all the sweeps (Fig. 9*E*). To reproduce the Adap, we include a spike-triggered small conductance K^+^ channel in our model (Fig. 8*I*) that generates after-hyperpolarization current reproducing current-independent adaptation, as in Liu and Wang (2001).

After a similar tuning of the VIP cell model, based on the phase diagram (Fig. 8*C*), the noise level (Fig. 8*M*), the jump of the Δ*b* (Fig. 8*F*), we can reproduce simultaneously the OB, which is reflected in the high onset and the fast-dropping of the firing rate during the first 40 ms (Fig. 8*J*,*N*), IR, which is reflected by the high IR score and high CV of the ISIs (Fig. 8*K*,*O*), and Adap, which shows exponentially decay during the latter 900 ms (Fig. 8*L*,*P*).

However, there are limitations to this VIP cell model. First, although the irregularity during the steady state is similar between the data and the model, the onset of IR firing in the model is usually later than that in the data. Especially when the injection current is around the rheobase, the IR firing may be observed after 1 s in the model (Fig. 9*A*). In contrast, in the data, it may be observed between 500 ms and 1 s (compare Fig. 8*B*). Second, the cell in the model becomes quiescence after the OB period (∼100 ms, Fig. 9*B*,*C*), which results from the strong Adap current triggered by several APs during the OB and the fast activation of the SIK current. But this is not the case in the data (Fig. 8*B*). In the model, this quiescence period diminishes when the injection current is high enough *I_inj_* = 237 pA (Fig. 9*C*). Since we are measuring the Adap starting at 100 ms, the diminishing of the quiescence period is reflected in the onset firing rate after 100 ms (Fig. 9*D*) and is further reflected in the jump on the AI curve (Fig. 9*E*).

To get an insight into what impacts firing after the OB period, we change the timescale of the fast activation variable of the SIK channel, which delays the onset of outward SIK current (Fig. 9*F*). As expected, the firing after the OB continues. But to achieve a comparable length with the data, the timescale needs to be ∼80 ms, which is unlikely for an SIK channel. We discuss this discrepancy further in the discussion section.

### Mechanism of IR firing in the VIP cell model

**Figure 10.**
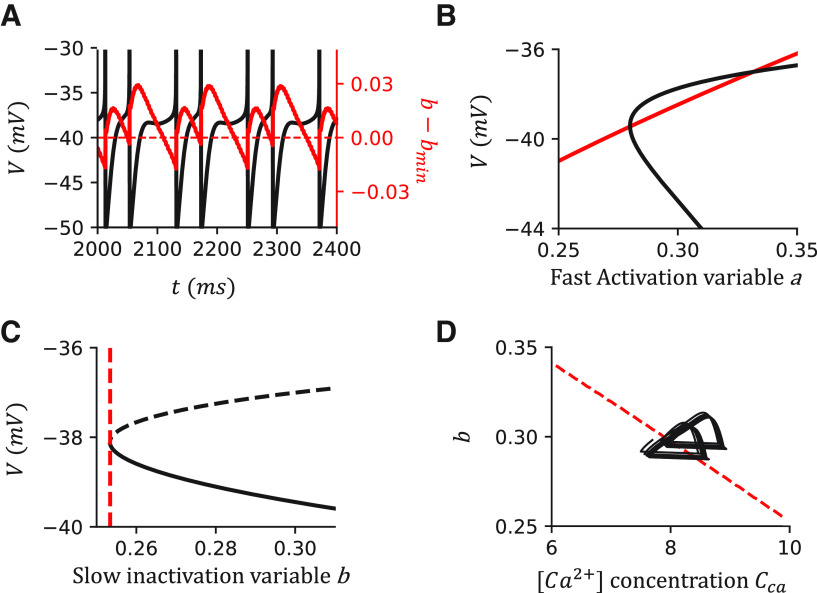
The fast-slow analysis of the VIP cell model shows that the irregularity results from the square-wave bursting. ***A***, The sample simulation when I_inj_ = 170 pA. Black line indicates the voltage trace. Red line indicates the difference between *b* and *b_min_*. Red dashed line is at *b* – *b_min_* = 0, suggesting the boundary between the resting state and the firing state. ***B***, Nullclines of the fast manifold when I_inj_ = 170 pA, *C_ca_* = 10, *b* = 0.3. The intersections indicate the fixpoints of the fast manifold. ***C***, The bifurcation diagram when I_inj_ = 170 pA, *C_ca_* = 10. Black solid line indicates the stable branch. Dashed line indicates the unstable branch. Red dashed line indicates the minimum of *b* that the fast manifold has a global SS. ***D***, The bifurcation diagram of the whole system and the simulation when I_inj_ = 170 pA. Red dashed line, b_min_(C_ca_), indicates the SNIC bifurcation line. Only the region above which has a global SS. Black line indicates the simulation of the VIP cell model without noise (σ = 0).

To investigate the origin of IR in the VIP cell model, we do a similar fast-slow analysis as we did for the canopy cell model. We first exclude the noise term in the model by setting σ = 0 and observe cluster spiking in the simulation (Fig. 10*A*). In contrast to the canopy cell model, we have two slow variables: slow inactivation variable *b* and the dimensionless Ca^2+^ concentration *C_ca_*. To do the fast-slow analysis, we assume both are constants. Similarly, the system has one SS and one US when either outward current prevails the injection current (Fig. 10*B*). By assuming *C_ca_* is a constant, we can define the *b_min_*(*C_ca_*) as the minimal *b* where a global SS exists (Fig. 10*C*, red dashed lines). In the full system, the line *b_min_*(*C_ca_*) represents the line where the SNIC bifurcation happens (Fig. 10*D*, red dashed lines). Only in the region above the bifurcation line, a global SS exists. In the simulation of the full model (Fig. 10*A*,*D*), the system oscillates between a resting state and a spiking state, which is represented as cluster spiking. The dynamics change to IR firing when adding noise to the system by setting σ > 0.

### Mechanism behind the traditional alias “IR spiking cell” for VIP cells

Traditionally, the term IR spiking has been used mainly for VIP cells but rarely for other INs (Tremblay et al., 2016). But, in our study, the irregularity is not uniquely found in VIP cells but distributed in multiple subtypes of L1 interneurons. Especially, the IR in the VIP cells is not because of a higher noise level. Indeed, the noise levels around the rheobase are comparable across all the subtypes (Figs. 3*G*, 5*F*, 7*E*, 8*M*). Is there anything unique for the IR VIP cells but not other INs with an IR feature?

**Figure 11.**
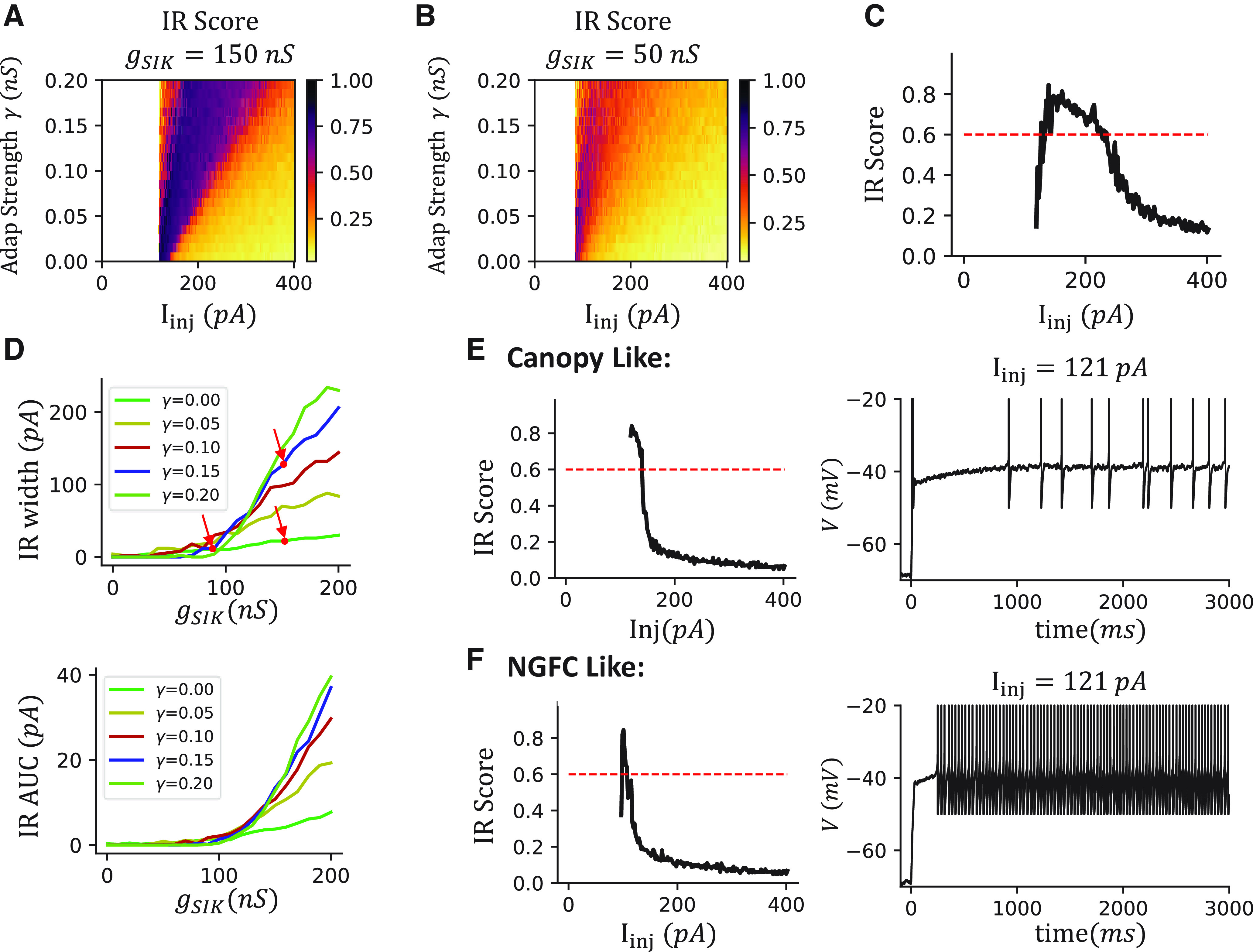
The IR firing of VIP cells is more easily observed in experiments because of a large IR width introduced by the interaction between *I_SIK_* and *I_adap_*. ***A***, The ISI ratio (color) over different injection currents and Adap strength γ when g_SIK_ = 150 nS. ***B***, Same as in ***A***, but g_SIK_ = 50 nS. ***C***, The sample ISI ratio when g_SIK_ = 150 nS, γ = 0.1. The range of injection current when CV ISI is above the threshold 0.6 is defined as the IR width. ***D***, The irregularity visibility is high only when the *g_SIK_* and γ are both large. Top, The IR width curves over *g_SIK_*. Different lines indicate results with different Adap strength γ. Dots and arrows indicate the parameters of the VIP cell model (top right), the canopy-like case (bottom right, shown in ***E***), and the NGFC-like case (left, shown in ***F***). Bottom, The IR area-under-curves, defined as the area under the curve of ISI ratio and above the ISI ratio threshold 0.6. ***E***, The model results when set γ = 0, g_SIK_ = 150 nS look like a canopy cell. Left, The IR score. The drop of the curve is sharp when increasing the injection current, and the IR width is small. Right, Voltage trace at I_inj_ = 121 pA. ***F***, The model results when setting γ = 0, g_SIK_ = 90 nS looks like an NGFC. The IR width is further reduced compared with ***A***. In this case, we remove the T-type CA^2+^ current for better visualization.

To answer this question, we systematically vary the parameters of our VIP cell model. We measure the IR score while varying Adap strength γ and the SIK conductance *g_SIK_* at different injection currents (Fig. 11*A*,*B*). The IR score is higher when the SIK conductance is bigger. Interestingly, the high IR score is more spread out with a stronger Adap. To quantify this effect, we define IR width as the range of injection current where the IR score is bigger than the threshold 0.6 (Fig. 11*C*). As a result, we observed that the IR width only starts increasing when *g_SIK_* reaches at ∼100 nS (Fig. 11*D*, top). However, the IR width remains small if an Adap current is not included γ = 0. The IR width only becomes large when the Adap strength is also increased. The results are similar if we use the IR area under the curve instead of IR width (Fig. 11*D*, bottom).

These explain the behavioral differences observed in the canopy, NGFC, and VIP models. In our VIP cell model, the IR width is at ∼120 pA (Fig. 11*D*, right top arrow; *g_SIK_* = 150 nS, γ = 0.1). When the Adap current is removed by setting γ = 0, the IR width decreases drastically to ∼20 pA (Fig. 11*D*, right bottom arrow), where the dynamics mimic the IR in canopy cells (Fig. 11*E*). If, in addition, we decrease SIK conductance to *g_SIK_* = 90 nS, the IR width decreases to ∼10 pA (Fig. 11*D*, left arrow), where the dynamics mimic the Acc in NGFC (Fig. 11*F*). These results suggest that IR is most easily observed only when the *g_SIK_* is big and the Adap is strong, which is the case for VIP cells, but not other INs.

To verify this claim in our dataset, we measured the IR width by multiplying the number of sweeps with an IR score bigger than 0.4 by the corresponding current step size of IR cells in different IN subtypes. The average IR width of VIP cells is the highest, and it is much higher than that of canopy cells (Table 2). Furthermore, the input resistance is the highest of the VIP cells compared with other L1 INs (Table 2), including α7 cells. Although the IR width of VIP cells and α7 cells is comparable, the corresponding voltage change of VIP cells is much higher than that of α7 cells, which explains why the IR feature is most salient in VIP cells.

### VIP cell model resonant with theta/alpha input through the activation of T-type Ca^2+^ channel

Because of the intrinsic properties, neurons may respond differently to an input of varied frequencies although the mean is the same. Theoretical and experimental studies suggested that these responses, which are referred to as the DG, are important in understanding the response from a neuron ensemble (Fourcaud-Trocmé et al., 2003; Geisler et al., 2005; Köndgen et al., 2008).

Recently, Borda Bossana et al. (2020) reported that rat L1 INs are homogeneous and narrow-tuned through the DG analysis by using a colored noisy input generated by an OU process, developed by Higgs and Spain (2009; see also Ilin et al., 2013). Briefly, in this analysis, a subthreshold colored noisy current signal, generated from an OU process with a time constant τ_σ_ = 5 ms, is given as input. Since the average input *I_inj_* is subthreshold, the responding APs are results of noise, but not a tonic, component of the input. The average current *I_inj_* and noise level σ are tuned such that the average firing rate is about <ν> = 5 Hz and the SD of voltage is ∼4 mV. These procedures are required to have similar membrane potential fluctuations that recorded *in vivo* (Ilin et al., 2013). Next, long recordings are made (at least 1000 s long and collecting 5000 APs), and further analyzed in the Fourier space. The ratio between the response and the input at a specific frequency is defined as the DG at that frequency (*DG*(*f*), referred to as the dynamic transfer function in Borda Bossana et al., 2020).

**Figure 12.**
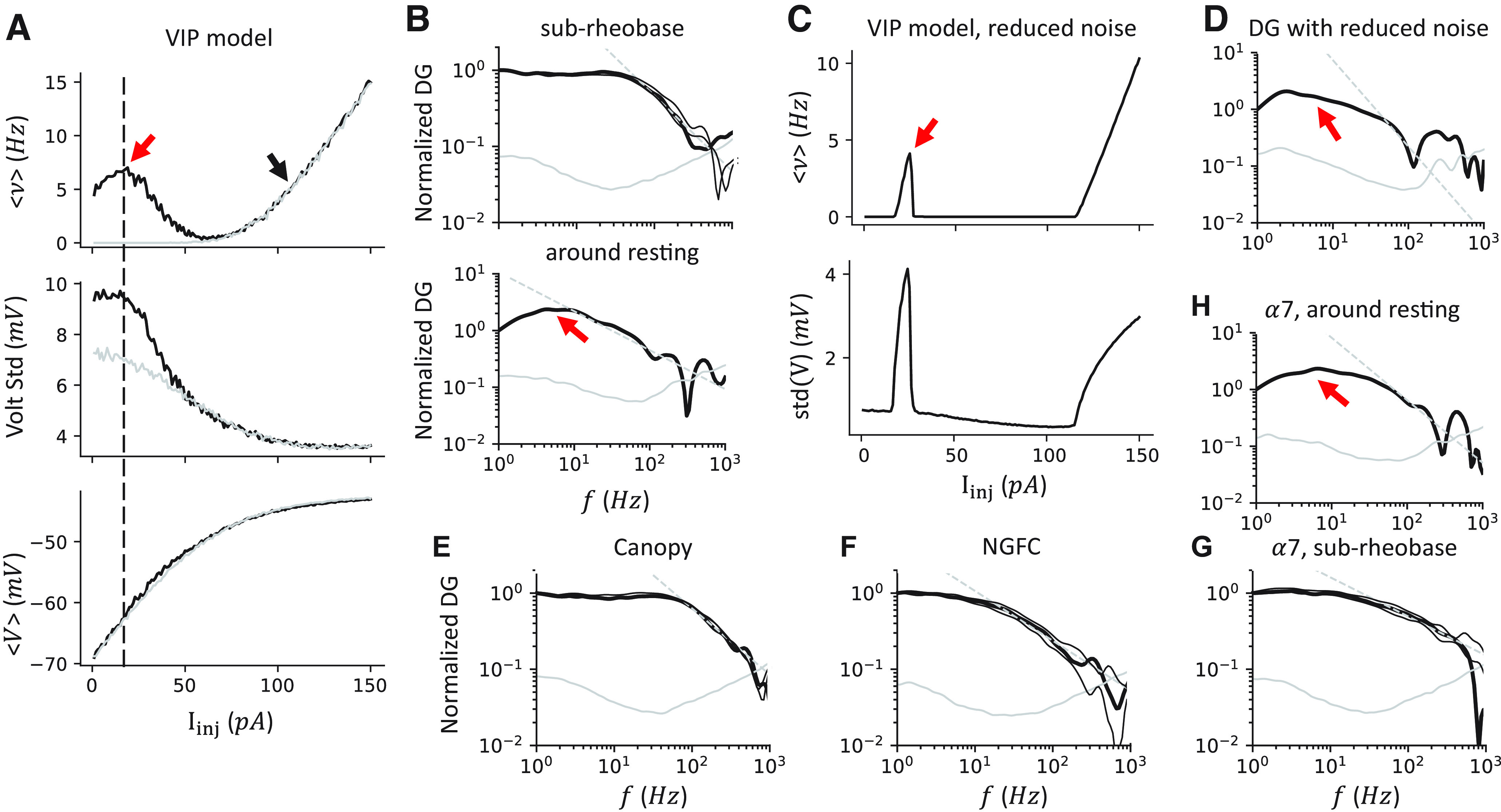
The VIP model resonates with theta/alpha band input only when the average input is around the resting. ***A***, Simulation results of the VIP model when varying the average injection current. In this panel, σ = 50 pA. Gray lines model results while excluding the T-type Ca^2+^ channels. Top, The average firing rate ν shows two non-zero branches. Middle, The voltage's SD decreases when the injection current increases. Bottom, The average voltage of the model. ***B***, The normalized DG when the injection current is at the sub-rheobase region (I_inj_ = 20 pA, black arrow in ***A***) and around the resting (I_inj_ = 105 pA, red arrow in ***A***). The DG shows an unexpected high-gain range around the theta/alpha band when the model is around the resting regimen. The high-frequency profile was described as *f*^–α^ with a cutoff frequency *f*_0_ and α, shown as a dashed gray line. The two thin black lines indicate the simulation with average firing rates <ν> = 3, 7 Hz, respectively. Solid gray line indicates the 95% confidence level generated by bootstrapping the shuffled ISIs. Top, *f*_0_ = 58.9 Hz, α = 0.99. Bottom, *f*_0_ = 67.6 Hz, α = 0.69. ***C***, Simulation results of VIP model with reduced noise σ = 5 pA. Top, The average firing rate. Bottom, The SD of the voltage. ***D***, The DG around the resting still shows a high gain range around the alpha band (I_inj_ = 25 pA, red arrow in ***C***) when the input noise is drastically reduced (*f*_0_ = 41.7 Hz, α = 1.52). ***E-G***, The normalized DG for the canopy cell model (I_inj_ = 282 pA, σ = 90 pA, *f*_0_ = 77.6 Hz, α = 0.62), NGFC model (I_inj_ = 170 pA, σ = 80 pA, *f*_0_ = 14.8 Hz, α = 0.65), and α7 cell model (I_inj_ = 200 pA, σ = 65 pA, *f*_0_ = 38.9 Hz, α = 0.51) when the average input current is at sub-rheobase. ***H***, The α7 cell model also has a non-zero firing branch around the resting that shows high gain around the theta/alpha band frequency (I_inj_ = 25 pA, σ = 65 pA, *f*_0_ = 83.2 Hz, α = 1.10).

Here, we test the response of our VIP cell model, along with other L1 IN models, to the different rhythm inputs through the DG analysis. We find that, with a designed synaptic input fluctuation (tuned at the black arrow in Fig. 12*A*), the VIP cell shows two non-zero firing rate branches (Fig. 12*A*). The sub-rheobase branch is expected where the APs are triggered by colored noise. However, when we decrease the average injection current while keeping the noise σ the same, the second branch around the resting voltage emerged, with an increased potential fluctuation and a peak ∼–60 mV. This second branch disappears if the T-type Ca^2+^ channel is removed from the model (*g_T_* = 0, Fig. 12*A*, gray lines). Within the sub-rheobase regimen (Fig. 12*B*, top), the DG shows a similar “narrow bandwidth” as reported (Borda Bossana et al., 2020). The results are robust when the average firing rate varies (3 and 7 Hz, Fig. 12*B*, top, two thin lines). Interestingly, around the resting regimen (Fig. 12*B*, bottom), the model resonates to the theta/alpha (4-15 Hz) band input. The observed resonance has characteristics that agree with the dynamic of the T-type Ca^2+^ channel. This resonance happens at the average voltage at ∼–60 mV, where the T-type Ca^2+^ channel activates (Fig. 12*A*, bottom). Also, the alpha/theta rhythm matches the activating time constant of the T-type Ca^2+^ channel τ^+^_h_ = 100 ms.

To make the voltage fluctuation comparable, we reduced the input noise σ, while the non-zero firing branch around the resting remains (Fig. 12*C*). Similarly, we observed high gain to the theta/alpha band input (Fig. 12*D*).

We further tested the DG in other IN models (Fig. 12*E–H*). All model shows a similar “narrow bandwidth” feature at the sub-rheobase regimen, suggesting the DGs of L1 IN models are homogeneous, as reported (Borda Bossana et al., 2020). Only the α7 model, which includes the T-type Ca^2+^ channel, shows another non-zero firing regimen around the resting, where the model resonates to the theta/alpha band input (Fig. 12*H*).

## Discussion

The neocortex is characterized by a remarkable diversity of GABAergic interneuron subtypes (Markram et al., 2004). Their functions have been proposed to serve diverse functions, exemplified by the well-established disinhibitory motif composed of three inhibitory subtypes (Wang et al., 2004; Tremblay et al., 2016). In this study, we systematically analyze the distribution of electrophysiological features in the L1 IN subtypes and further build models for each subtype by including different ion channels. Canopy cells and NGFCs mostly show IR firing or acceleration in the firing rate, suggesting SIK exists in these two cell types. Most of the α7 cells are classified as having an OB feature, which implies the presence of a T-type Ca^2+^ channel. The VIP cells are very heterogeneous, but many show Adap and IR firing. To reproduce that, we build a model that includes an SIK channel, a T-type Ca^2+^ channel, and a spike-triggered Ca^2+^-dependent K^+^ channel. We apply slow-fast analyses to show that the irregularity comes from the square-wave bursting, which requires a strong SIK current. Further, we showed that the Adap current observed within the VIP cells significantly expands the region where the IR firing can be observed, and this is the main reason why VIP cells are often considered IR spiking cells but not other INs (Tremblay et al., 2016).

Our work provides insights into outstanding questions concerning interneuron subtype biology. First, by revealing the mechanism of IR firing of VIP cells, applying an antagonist to either block the SIK channel or the Adap current should eliminate the IR firing. Applying gene-editing methodology in cultured cells should show similar results as well. Second, our VIP model suggests that the different electrophysiology subtypes from VIP cells may come from continually varying the strength of different ion currents, thus contributing to the classification of VIP subtypes. For instance, the cells classified as IS, fAD, and bNA in He et al. (2016) may be reproduced by just including a strong SIK current, a strong T-type Ca^2+^ channel with or without a strong Adap current, and a combination of T-type Ca^2+^ current with an SIK current, respectively. Third, we revealed an unexpected resonance of alpha/theta band input in the VIP cell model and α7 cell model around the resting voltage, which can be measured experimentally. Previously (Higgs and Spain, 2009), the bursts generated from the fast afterhyperpolarization provide a high gain at a similar rhythm band (7-16 Hz). Similarly, our model predicts that the alpha/theta resonance is associated with the OB, and both features arise from the dynamics of the T-type Ca^2+^ channel.

Detailed reconstructed models of rat L1 INs, along with every neuron subtype, were studied in the Blue Brain Project (BBP) (Markram et al., 2015) through a systematically optimizing algorithm. Within their study, most L1 INs are classified as continuous nonaccommodating cells, while only a few are stuttering or IR firing. However, only a limited number of sweeps (1.5×, 2×, 2.5× rheobase) are used in classification and further optimizing their models. In our dataset, canopy cells and NGFCs account for ∼70% of the total L1 INs population, and most are classified as having an IR or an ACC feature. This does not contradict the BBP observation since we consider the finer recordings around the rheobase. If we only consider the recordings away from the rheobase, the NFGCs and canopy cells would be classified as continuous nonaccommodating cells as BBP did.

Further, the BBP models and our model differ in reproducing IR firing. The BBP models introduce the high channel noise from a stochastic potassium channel to reproduce the irregularity (Markram et al., 2015, their supplementary materials, Optimization of neuron models). However, we showed that this is unnecessary if an SIK channel is included. Indeed, our data show that the noise level is comparable between IR firing cells and others (Figs. 3*G*, 5*F*, 7*E*, 8*M*), which suggests that high channel noise is not the reason for the IR firing.

We build our models based on an EIF model with a few additional ion channels to reproduce the major observed dynamics. Thus, the models are easy to analyze, and the computational cost is minimized when implemented in a large-scale neuronal circuit, contrasting to the detailed reconstructed multicompartmental models (for examples, see Markram et al., 2015). However, doing so limits the ability to reproduce electrophysiological features from various aspects. Unlike Hodgkin-Huxley type models, our model does not include the dynamics during the APs. Thus, we cannot reproduce the features of APs from different subtypes (Table 2) in our model. Another issue is that we mostly reproduce the firing behavior around the rheobase (<20 Hz), but not with high firing rates. This is because our dataset includes recordings mostly around the rheobase but not with high firing frequency (most recordings have < 30 APs), and our EIF model, unlike the Hodgkin-Huxley type model that stops firing when the outward currents are overwhelmed by the injection current, can have arbitrary high firing rates. In addition, the f-I slope in our model is higher than that measured from the data. From our experience, only the capacity significantly impacts the f-I slope in the model, which is constrained (Fig. 2; Table 2). We speculate that this mismatch can be alleviated by including other outward currents, like K^+^ current from KCNQ channels (Goff and Goldberg, 2019).

Late-spiking is a signature of neurogliaform cells, but the underlying mechanism is unknown. In delayed-firing hippocampal CA1 pyramidal neurons (Storm, 1988), blocking Kv1, an SIK current, with low concentrations of 4-aminopyridine (4-AP), eliminates the delayed-firing feature. Further, in delayed fast-spiking cells, a dendrotoxin-sensitive Kv1 current, underlies the delayed firing (Goldberg et al., 2008; but see also Bos et al., 2018) and clustered spiking with subthreshold oscillations (Sciamanna and Wilson, 2011). Based on these experiments, we include an SIK channel in our NGFC model and reproduce the late-spiking and firing-rate acceleration. However, a recent study on NGFCs suggested that, although Kv1 currents exist in the mouse NGFCs and contribute to the delayed-firing (Chittajallu et al., 2020, their Fig. 4I), applying low 4-AP decreases ∼65% of the AP latency), their functional role may be lesser than that of other K^+^ currents, especially from the Kv4 family. Furthermore, preliminary results from our laboratory suggest that dendrotoxin does not block delayed firing in NGFCs. Interestingly, the recovery time from inactivation is ∼1 s (Chittajallu et al., 2020) and ∼12 s (Bos et al., 2018), which is slower than that in our model (∼500 ms, Fig. 6*C*). Nevertheless, this discrepancy is not likely explained by the dynamics of Kv4 channels, which are faster than that of Kv1 channels (Coetzee et al., 1999). Further pharmacological experiments are necessary to help discover the slowly inactivating K^+^ current of NGFCs.

A significant difference between NGFCs and canopy cells is that NGFCs are late-spiking, whereas canopy cells are not. However, if canopy cell models only include an SIK channel, they are also late-spiking (Fig. 3*M*). Also, VIP cells often show decreased firing rates after the OB period, usually at ∼100 ms, but are not quiescent (Fig. 8*B*). This behavior was hard to reproduce in the model because of the large Adap current triggered by several APs in the OB period. These discrepancies in the canopy model and the VIP model suggest the existence of transient inward currents (or inactivation of outward currents) that have slower dynamics than the T-type Ca^2+^ current. In VIP cells (Goff and Goldberg, 2019), the Nav1.1 channel may contribute to the length of the onset firing period via progressive inactivation during repetitive firing. Further studies are required to validate the role of Nav1.1 channels in models with an IR feature.

Recently, it has become clear that L1 is the key to understanding how long range cortical–cortical interactions alter local circuit dynamics (Ledderose et al., 2021; Schuman et al., 2021). For example, recent work in rodents showed that hippocampal-dependent associative learning is controlled by perirhinal input to L1 (Doron et al., 2020; Shin et al., 2021). More importantly, this associative learning could be diverted by dendritic inhibition. Another recent study had shown that L1 NDNF^+^ INs (NGFCs and canopy cells in our study) can shift the input gain from dendrite to soma in pyramidal cells across all layers in the cortex (Cohen-Kashi Malina et al., 2021), suggesting that L1 NDNF^+^ INs may be well suited to control associative learning. Further, another anatomic study showed that ventromedial and mediodorsal thalamus target NDNF^+^ cells and VIP^+^ cells in mouse PFC, respectively (Anastasiades et al., 2021), through which these higher-order thalamic nuclei differentially modulate the neuronal activity in the PFC. To further investigate the hypotheses listed here and beyond, a well-developed model that incorporates the distinct properties of each L1 interneuron subtype will help in elucidating the mechanisms by which the L1 circuit enables the integration of top-down and bottom-up information streams, and will assist in the generation of predictions to guide future experiments. Our work on the individual L1 IN subtypes here is an essential step toward this goal.
